# The use of wearable resistance and weighted vest for sprint performance and kinematics: a systematic review and meta-analysis

**DOI:** 10.1038/s41598-024-54282-8

**Published:** 2024-03-05

**Authors:** Gabriel Felipe Arantes Bertochi, Márcio Fernando Tasinafo Júnior, Izabela A. Santos, Jeffer Eidi Sasaki, Gustavo R. Mota, Gabriela Gregorutti Jordão, Enrico Fuini Puggina

**Affiliations:** 1 Graduate Program in Rehabilitation and Functional Performance, Ribeirao Preto Medical School, University of Sao Paulo, Ribeirao Preto, SP Brazil; 2 School of Physical Education and Sport of Ribeirao Preto, University of Sao Paulo, Ribeirao Preto, SP Brazil; 3 Exercise Science, Health and Human Performance Research Group, Department of Sport Sciences, Institute of Health Sciences, Federal University of Triangulo Mineiro, Uberaba, MG Brazil; 4 Institute of Health Sciences, Department of Nutrition, Federal University of Triangulo Mineiro, Uberaba, MG Brazil

**Keywords:** Anatomy, Musculoskeletal system

## Abstract

Wearable resistance (WR) and weighted vests (WV) can be used in almost all training conditions to enhance sprint performance; however, positioning and additional mass are different in WV and WR strategies, affecting performance and kinematics differently. We aimed to systematically review the literature, searching for intervention studies that reported the acute or chronic kinematic and performance impact of WV and WR and comparing them. We analyzed Pubmed, Embase, Scopus, and SPORTDiscuss databases for longitudinal and cross-over studies investigating sprint performance or kinematics using an inverse-variance with a random-effect method for meta-analysis. After the eligibility assessment, 25 studies were included in the meta-analysis. Cross-over WR and WV studies found significantly higher sprint times and higher ground contact times (CT) compared to unloaded (UL) conditions. However, WR presented a lower step frequency (SF) compared to UL, whereas WV presented a lower step length (SL). Only one study investigated the chronic adaptations for WR, indicating a superiority of the WR group on sprint time compared to the control group. However, no difference was found chronically for WV regarding sprint time, CT, and flight time (FT). Our findings suggest that using WV and WR in field sports demonstrates overload sprint gesture through kinematic changes, however, WR can be more suitable for SF-reliant athletes and WV for SL-reliant athletes. Although promising for chronic performance improvement, coaches and athletes should carefully consider WV and WR use since there is no supporting evidence that WV or WR will impact sprint performance, CT, and FT.

## Introduction

Sprinting is a fundamental skill in many athletic disciplines and team sports, as faster individuals can have a decisive advantage in sprint placements and performance during sporting requirements^1,2^. Plyometric and heavy weight training, despite being fairly traditional strategies, are of limited transfer to sprint performance, leading to minimal improvement (ranging from − 0.65% to − 4.3%) in sprinting sports gestures, even with up to 44% strength improvement^3,4^. Furthermore, these training methods may exhibit inconsistent outcomes and be time-consuming^5^. Consequently, specific training strategies, like regular sprint training, have been the first options to enhance short-distance sprinting performance based on the specificity principle^6^.

Thus, resisted/loaded sprint strategies, such as sled sprinting, Bulgarian bag, and weighted vests (WV), are increasingly being used due to their potential to provide high-specificity overload during sprint training, aiming to enhance performance^7,8^. However, since they demand larger equipment and space, sled sprinting and Bulgarian bags may not be feasible in all training conditions, such as tactical training for team sports. Thus, WV is a more viable strategy for promoting specific overload during sprinting training and can be used in most conditions and training environments. Recently, a newer technology (i.e., Lila™ Exogen™ *compression shorts*) enabled WV to be used with different distributions and weight manipulation around the body, called wearable resistance (WR)^9^.

The higher body mass (BM) provided by these resources, such as WV and WR, can directly impact kinematics during sprinting. A decreased velocity, increased ground contact time, decreased step frequency, and decreased step length represent a variety of kinematic outcomes that can be presented depending on the load magnitude, load location, and type of clothing (WV or WR) used when sprinting^10–14^. These kinematic changes highlight the overload capacity of vesting strategies. Thus, recently, many studies have been conducted to investigate the acute and chronic impact on performance and kinematic aspects of these techniques^10,12,15–17^.

However, WV is traditionally used in the trunk, enabling larger loads when compared to WR. It is common to find studies that have used loads exceeding 10% of BM through WV^18,19^. On the other hand, WR has been traditionally used in the limbs, such as the forearm, shank, and thigh^16,20,21^. The loads reported in WR are normally lower than 5% of BM and can produce higher rotational inertia in the limbs^9^. In this sense, it is clear that WV applied to the trunk will impact performance and kinematics differently from WR. Thus, this systematic review and meta-analysis aimed to report the kinematic and performance impact of WV and WR and compare them.

## Methods

This systematic review and meta-analysis followed the Preferred Reporting Items for Systematic Reviews and Meta-Analyses (PRISMA) criteria.

### Eligibility criteria

Eligibility was assessed using the PICOT strategy. Studies were included if they had included adult athlete individuals (P); analyzed sprint performance or kinematics, with any form of additional wearing load with the total load and segment of the body reported. Both analyses must have been performed in maximal sprint tests (I); had the presence of a control group (CON) or unloaded condition (UL) (C); reported the influence of additional wearing load in step frequency or step length or flight time or ground contact time or sprint time (O); conducted an experimental cross over or longitudinal study (T). We included studies in English, Spanish, and Portuguese language. Case studies were not included. No data filter was used.

### Information sources and search strategy

The databases used were PubMed, Embase, Scopus, and SPORTDiscuss with the following search strategy: (“Wearable resistance” OR WR OR “mass manipulation” OR “extra-load*” OR “extra load*” OR “added load*” OR “limb load*” OR “arm load*” OR “leg load*” OR “thigh load*” OR “shank load*” OR hypergravity OR “weighted vest*” OR “hypergravity condition*” OR “resisted sprint*”) AND (sprint* OR “sprint run*” OR running OR “sprint acceleration” OR “sprint kinematics” OR “sprint speed” OR “sprint performance” OR “sprint-running”). We used the “title, abstract, keywords” filter for Scopus and Embase.

### Selection and data collection process

The search results were uploaded to Rayyan Software^22^. Firstly, the duplicates were identified automatically (https://www.rayyan.ai/). Two independent authors (GFAB and IAS) conducted the initial screening for the title and abstract based on the PICOT strategy. The articles selected were read in full. Articles that met all the eligibility criteria were included. The first search was performed on 05/11/2022 and then updated on 08/30/2023.

### Data items

The included articles were first separated into WR or WV interventions to compare both conditions. Then, cross-over and longitudinal studies were separated for further subgroup analysis in WR loads (≤ 2%; > 2%), WR placement (upper body; lower body), and sprint phase (acceleration; maximum velocity).

For the studies that did not report the WR/WV load in % of BM, the load was normalized by the average BM of the sample. Acceleration and maximum sprint phases were defined as equal or less than 30 m and more than 30 m, respectively^20,23^. The variables of interest were kinematic data, sprint time, sample size, age, height, BM, training experience, best sprint performance, study design, ability level, study intervention, objective, sprint test, WR/WV load and placement.

### Study risk of bias and quality assessment

Two independent authors (IAS and MFTJ) assessed the quality of the included articles using the Physioterapy Evidence Database (PEDro) tool. The tool is composed of eleven criteria. Each criterion satisfied received 1 point (except for the first criterion). Articles scoring six to ten were considered high quality; scores between four and five were considered moderate quality; scores lower or equal to three were considered low quality. A senior author resolved discrepancies among authors (EFP). The publication bias was assessed visually from funnel plots for two authors independently (IAS and MFTJ); more asymmetry from the vertical axis can indicate publication bias^24^.

### Effect measures and synthesis methods

All analyses were conducted in the software Review Manager 5.3. An inverse-variance with a random-effect method was used. For longitudinal studies, the mean for the experimental and CON group was calculated based on the mean difference of the post-intervention value minus the pre-intervention value. The standard deviation was calculated from the pooled standard deviation of change scores in both groups by using a correlation value of 0.5 and the formula described $SD_{\text{E,change}} = \sqrt {{SD_{\text E,baseline}^{\text{2}}} + SD_{\text E,final}^{\text{2}} - \left( {2 \,X \,Corr \,X SD_{\text{E,baseline}} X \,SD_{\text{E,final}}} \right)}$ in the Cochrane Handbook for Systematic Reviews of Interventions^25^. For cross-over studies, authors used the mean and SD of the sample in the loaded condition as experimental mean and SD, and the same was done for the UL condition as control mean and SD. Only the total time of the performance tests was used (the split times were not considered) avoiding inflate the meta-analysis. In addition, the thigh outcome was chosen for the WR studies in which the effect of using the thigh or shank WR was verified, as it is the most common. The highest weight reported was considered for studies where more than one weight for WV and WR were tested. After that, the included studies were separated by intervention period (cross-over or longitudinal). Then, the following subgroup analysis was conducted for WR cross-over interventions: loads (≤ 2%; > 2%), placement (upper body; lower body) and sprint phase (acceleration; maximum velocity). Due to the lack of data, subgroup analyses were not done for WR and WV longitudinal interventions. The confidence interval used was 95% for a random effect model. For the heterogeneity analyses, the I^2^ test was used. The *P* ≤ 0.05 was considered statistical significance, and I^2^ > 50% indicated high heterogeneity.

For studies that only displayed data in figures, authors used the WebPlotDigitizer v. 4.6 (https://automeris.io/WebPlotDigitizer)^26^ to extract values.

## Results

### Study selection

**Figure 1:**
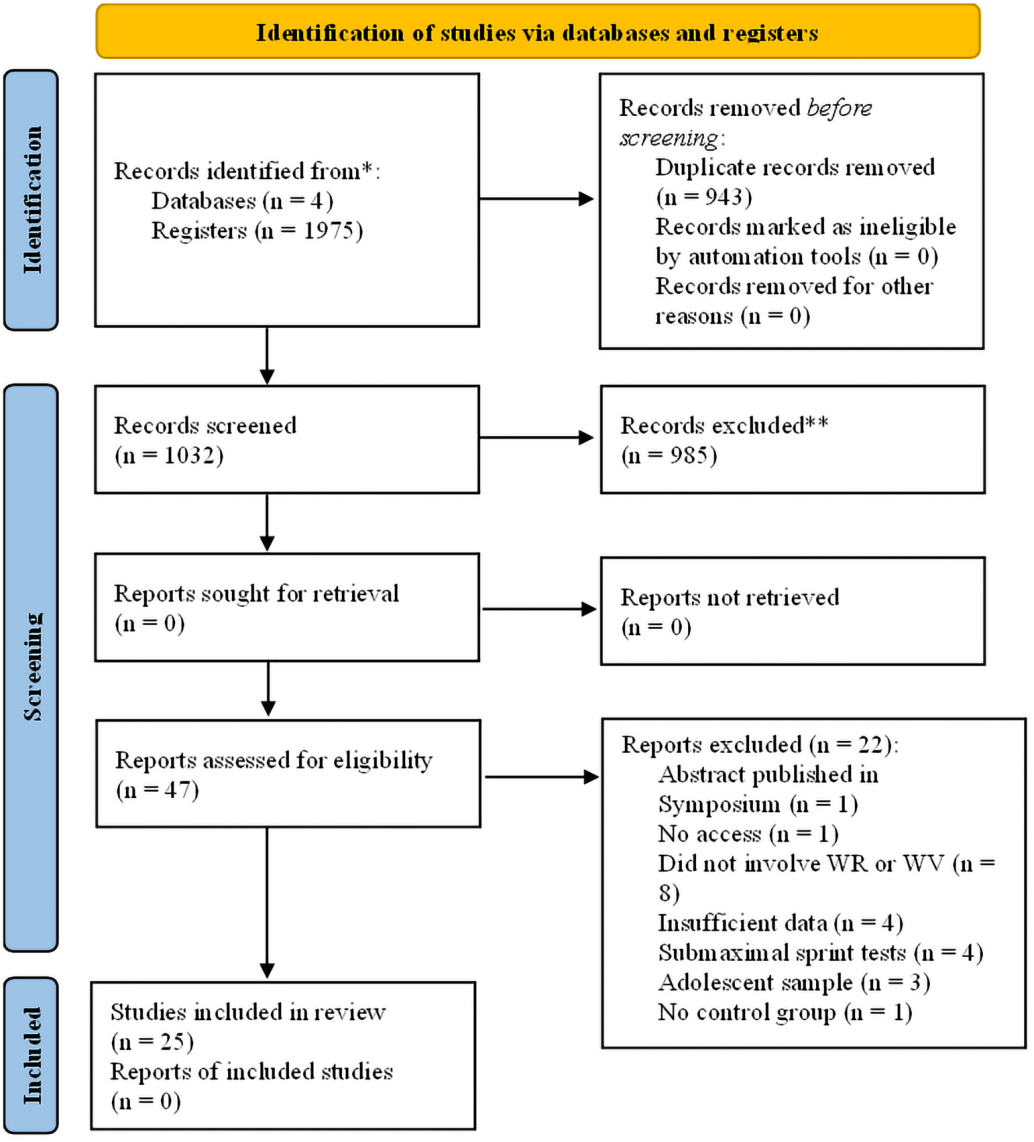
PRISMA Flow diagram.

Initially, 1975 studies were returned following the search strategy. On Embase, 401 studies were identified, 749 on Scopus, 405 on SPORTDiscuss, and 420 on PubMed. The authors removed 943 studies considered duplicates, resulting in 1032 studies included for the title and abstract screening. Of the 1032 records screened, 985 were considered ineligible, resulting in 47 studies in the last screening process. From the 47 studies read in full, one was excluded because only the abstract was published, one study was not accessible, eight studies did not involve WR or WV, four studies did not provide sufficient data to be included in the meta-analysis, four studies did not include maximal sprint tests, three studies were based on youth sample, and one study did not include a CON group. Thus, 25 studies were included in the systematic review and meta-analysis^10–21,23,27–38^. Figure 1 shows the study selection process.

### Study characteristics

The 25 studies totaled 406 participants (samples ranging from 7 to 24). The mean age, height, and BM of the participants from all studies were 21.47 years (ranging from 18 to 26), 176.46 cm (ranging from 160 to 186), and 76.51 kg (ranging from 63 to 97), respectively. Training experience varied from 2 to 14.7 years. Personal best sprint performance varied from 10.97 to 11.46 s (100 m). Fifteen studies involved WR in a cross-over design^14,16,17,17,20,21,23,27,29,31–34,36,38^. Five studies involved WV in a cross-over design^11,12,28,30,37^. One study involved WR in a longitudinal design^15^. Four studies involved WV in a longitudinal design^10,18,19,35^. The intervention period for longitudinal studies varied from 8 days to 7 weeks^10,15,18,19,35^. Ten studies reported a sample of sprinters ^12,20,23,27–30,32,36,38^. Seven studies reported a sample of rugby players^10,12,13,15,17,28,34^. Three studies reported the training status without mentioning the sport^14,16,35^. Two studies reported a sample of soccer players^11,19^. Two studies reported a sample of male athletes from university athletic clubs^31,33^. Lacrosse players, field hockey, miscellaneous sports, sport science students, and track and field athletes were reported only in one study each^12,18,21,37^. According to the PEDro scale, three studies were considered low-quality^23,27,30^, twenty-one were considered moderate-quality^10–18,20,21,28,29,31–38^, and one was considered high-quality^19^. The visual analysis of the funnel plots indicates a low risk of bias for the studies included. Characteristics for each study are presented in Table 1. Funnel plots for each comparison are presented in the Electronic Supplementary Material (Figure S1 to S13).

**Table 1:** Sample characteristics.

Study	Sample size	Age (years)	Height (cm)	Mass (kg)	Training experience (years)	Best sprint performance (s)	Design (intervention period)	Sport and ability level	PEDro
Barr et al.^10^	15 (WV = 8; UL = 7)	WV = 22.4 ± 2.7; UL = 22.0 ± 2.1	WV = 182 ± 6; UL = 186 ± 7	WV = 95.3 ± 7.1; UL = 92.8 ± 11.4	NA	NA	Longitudinal (8-day)	Training athletes from the national rugby team academy	5
Bennett et al.^27^	8	26.0 ± 7.3	177 ± 3	77.3 ± 3.9	Sprint-specific = 4.9 ± 1.4; Resistance training = 6.8 ± 8.1	11.1 ± 11.10 (100-m)	Cross-sectional	Male beach sprinters athletes	3
Carlos-Vivas et al.^11^	23	20.8 ± 1.5	180 ± 6	75.3 ± 7.3	NA	NA	Cross-sectional	Semi-professional soccer players	5
Clark et al.^18^	20 (UL = 7; WV = 6)	WV = 19.7 ± 0.9; UL = 19.9 ± 1.0	WV = 182 ± 8; UL = 178 ± 6	WV = 79.1 ± 5.2; UL = 81.7 ± 8.0	NA	NA	Longitudinal (7 weeks)	Lacrosse players	5
Cronin et al.^28^	20	19.9 ± 2.2	176 ± 8	76.5 ± 10.7	NA	NA	Cross-sectional	Track sprinters, beach sprinters, and rugby players	4
Cross et al.^12^	13	22.9 ± 3.3	179 ± 6	82.5 ± 8.4	NA	NA	Cross-sectional	Track sprinters, rugby, field hockey, and miscellaneous players	5
Feser et al.^20^	15	21.1 ± 2.22	174 ± 5	67.2 ± 4.5	9.33 ± 2.74	11.44 ± 0.42 (100-m)	Cross-sectional	University-level male sprint specialists	4
Feser et al.^15^	22 (WR = 10; UL = 12)	WR = 22.6 ± 2.9; UL = 24.6 ± 2.9	WR = 182 ± 8; UL = 178 ± 5	WR = 96.5 ± 13.6; UL = 92.5 ± 12.9	NA	NA	Longitudinal (6-week)	Collegiate/semi-professional rugby athletes	5
Feser et al.^29^	11	21.2 ± 2.56	175 ± 5	69.1 ± 3.85	Sprint training = 9.73 ± 2.90	11.34 ± 0.41 (100-m)	Cross-sectional	University-level sprint specialists	4
Feser et al.^38^	18	20.9 ± 2.05	174 ± 5	66.3 ± 5.06	9.17 ± 2.57	11.46 ± 0.40 (100-m)	Cross-sectional	University-level sprinters	5
Gleadhill et al.^30^	14	19.9 ± 1.2	173.9 ± 6.6	68.8 ± 4.4	NA	11.15 ± 0.33 (100-m)	Cross-sectional	Male sprinters regional-national level	3
Hurst et al.^23^	7	21 ± 1	173 ± 9	71.1 ± 6.6	NA	Male = 11.61 ± 0.39; Female = 12.0	Cross-sectional	University-level sprinters	3
Macadam et al.^31^	15	21 ± 2.5	174 ± 4.1	67.5 ± 5.4	NA	11.3 ± 0.5 (100-m)	Cross-sectional	Male athletes from university athletic clubs	4
Macadam et al.^32^	14	20.6 ± 1.2	172.8 ± 3.9	65.3 ± 4.9	NA	11.40 ± 0.39 (100-m)	Cross-sectional	Male collegiate sprinters	4
Macadam et al.^33^	15	20.9 ± 2.2	174.4 ± 5.1	66.8 ± 5.5	9.4 ± 2.6	11.40 ± 0.40 (100-m)	Cross-sectional	Male athletes from university athletic clubs	4
Macadam et al.^16^	14	24.9 ± 4.2	172.1 ± 6.8	68.4 ± 7.1	NA	NA	Cross-sectional	Recreational active subjects	4
Macadam et al.^34^	19	19.7 ± 2.3	181 ± 6.5	96.1 ± 16.5	At least two years of sprint experience	NA	Cross-sectional	Male amateur/semi-professional rugby athletes	4
Macadam et al.^17^	22	19.4 ± 0.5	180.4 ± 7.2	97 ± 4.8	NA	NA	Cross-sectional	Male amateur rugby union players	5
Rey et al.^19^	19 (WV = 10; UL = 9)	WV = 23.6 ± 2.7; UL = 23.7 ± 2.1	WV = 178.5 ± 4.9; UL = 179.8 ± 4.8	WV = 73.9 ± 6.5; UL = 75.1 ± 6.8	Soccer experience = 14.7 ± 4.1	NA	Longitudinal (6-week)	Male amateur soccer players	8
Simperingham et al.^13^	15	19.0 ± 0.5	181.2 ± 7.3	91.0 ± 17.4	NA	NA	Cross-sectional	Male rugby union athletes	4
Simpson et al.^35^	19 (WV = 9; UL = 10)	WV = 21 ± 2; UL = 22 ± 3	WV = 170 ± 3; UL = 160 ± 4	WV = 67 ± 4; UL = 63 ± 7.8	NA	NA	Longitudinal (3 weeks)	Trained females	5
Uthoff et al.^21^	14	20.61 ± 1.16	173 ± 3.85	65.33 ± 4.86	NA	11.40 ± 0.39 (100-m)	Cross-sectional	Recreational track and field male athletes	4
Uthoff et al.^36^	14	20.6 ± 1.16	172.8 ± 3.85	65.3 ± 4.86	NA	11.40 ± 0.39 (100-m)	Cross-sectional	Sub-elite male sprinters	4
Zafeiropoulos et al.^37^	24	18–23	178 ± 0.05	74.2 ± 8.9	With 2 years of soccer training experience	NA	Cross-sectional	Sport science students	4
Zhang et al.^14^	16	21 ± 2	176 ± 4	67.41 ± 5.72	NA	10.97 ± 0.34 (100-m)	Cross-sectional	Well-trained male athletes	5

Data are mean ± standard deviation.

NA: Not available; UL, Unloaded; WR, Wearable resistance; WV, Weight vest.

### Results of individual studies and syntheses

#### Cross-over WR interventions

Fifteen studies presented a cross-over design with a WR intervention^13,14,17,17,20,21,23,27,29,31–34,36,38^. Loads applied varied between 0.55 to 5% of the sample BM^13,14,17,17,20,21,23,27,29,31–34,36,38^. The most frequent load used was 2% in ten studies^16,17,20,21,29,31–33,36,38^, followed by 3% in three studies^13,16,34^. Sprint test distance varied between 10- and 65 m, with a study performing a 10-s maximum effort test^13,14,17,17,20,21,23,27,29,31–34,36,38^. The most frequent sprint test used was 30- and 50-m in four studies each^13,20,21,29,31–33,36^. Nine studies reported WR attached to the thigh ^13,16,20,23,27,31,33,34,38^, eight studies reported WR attached to the shank^13,14,20,23,27,29,34,38^, and four studies reported WR attached to the forearms^17,21,32,36^.

##### Sprint performance WR

**Figure 2:**
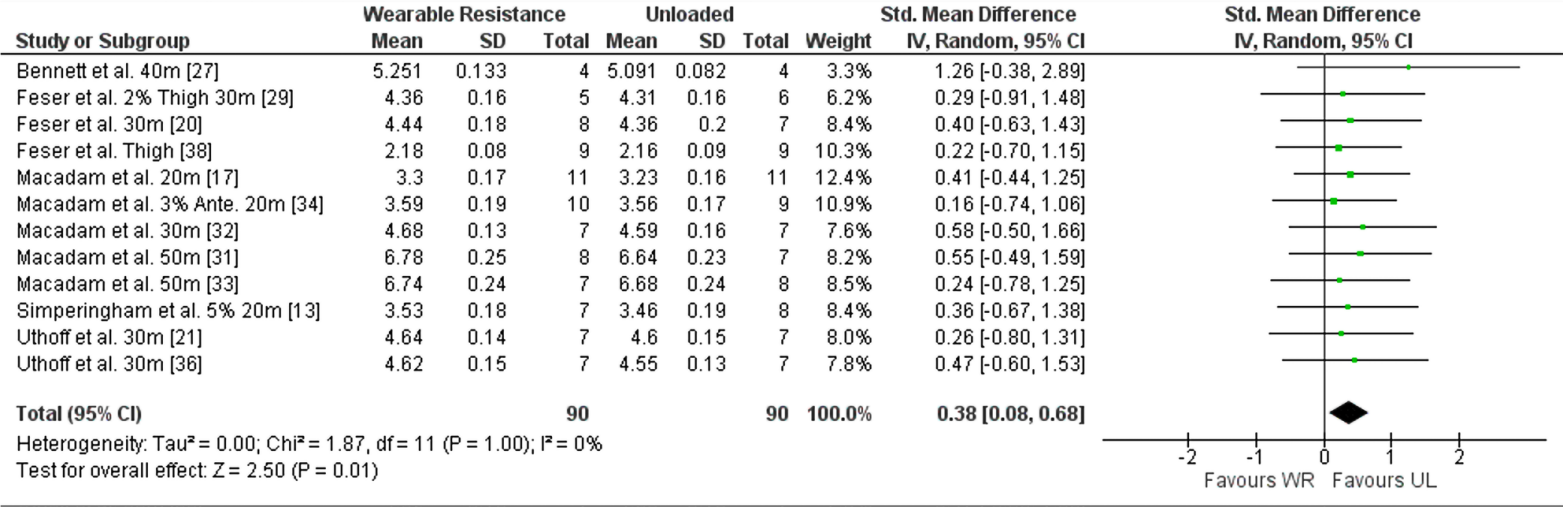
Forest plot of meta-analysis of the standardized mean difference from WR and UL conditions for sprint time. Ante., Anterior; CI, Confidence interval; SD, Standard deviation; UL, Unloaded condition; WR, Wearable resistance condition.

Results in meta-analysis indicated a significantly lower sprint time for UL condition (standardized mean difference [SMD] 0.38 (95% CI 0.08 to 0.68), Z = 2.50 (*p* = 0.01)) with a low, non-significant, average heterogeneity (I^2^ = 0% (*p* = 1.00)). The forest plot for meta-analysis is presented in Fig. 2. Twelve studies reported the sprint time^13,17,20,21,27,29,31–34,36,38^, with a 0 to 7.4% higher sprint time in the WR condition compared to the UL condition. Of those, ten studies reported the effect size (ES) for sprint time^13,17,21,29,31–33,36,38^, ranging from − 0.54 to 0.64.

For subgroup analysis, sprint time for the acceleration phase (SMD 0.34 (95% CI 0.01 to 0.67), Z = 2.01 (I^2^ = 0%)), and WR loads ≤ 2% (SMD 0.41 (95% CI 0.08 to 0.74), Z = 2.44 (I^2^ = 0%)), were significantly (*p* < 0.05) lower in UL condition compared to WR condition.

##### Ground contact time WR

**Figure 3:**
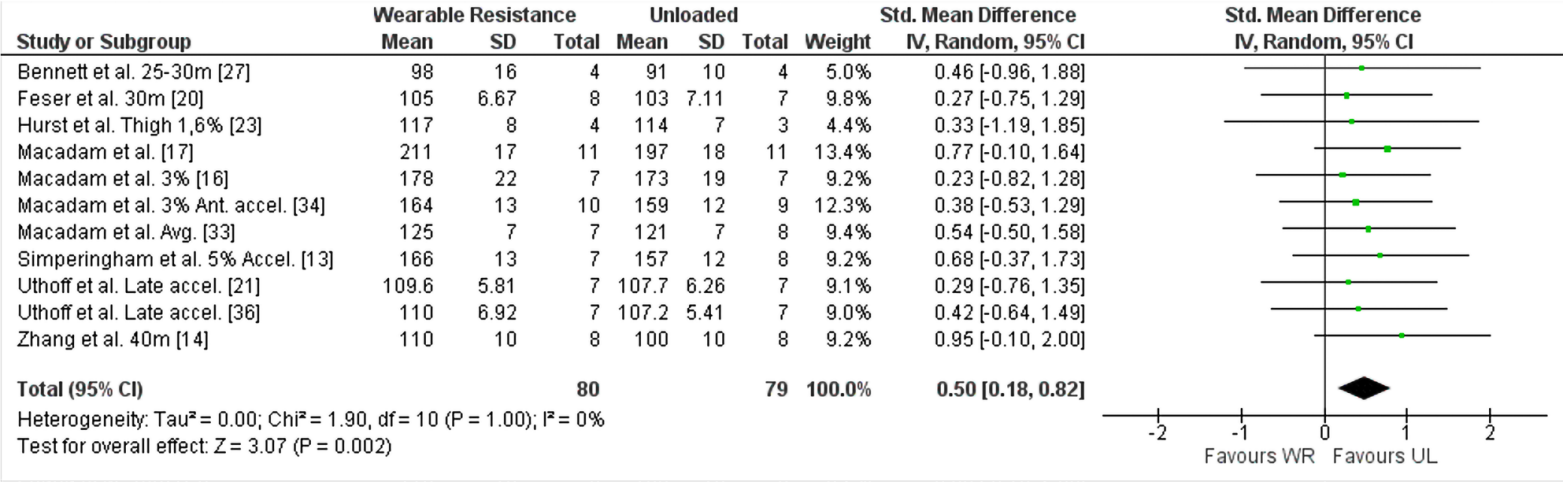
Forest plot of meta-analysis of the standardized mean difference from WR and UL conditions for ground contact time. accel., Acceleration phase; Ante., Anterior; CI, Confidence interval; SD, Standard deviation; UL, Unloaded condition; WR, Wearable resistance condition.

Results in meta-analysis indicated a significantly lower ground contact time for UL condition ([SMD] 0.50 (95% CI 0.18 to 0.82), Z = 3.07 (*p* < 0.01)) with a low, non-significant, average heterogeneity (I^2^ = 0% (*p* = 1.00)). The forest plot for meta-analysis is presented in Fig. 3. Eleven studies reported the ground contact time^13,14,16,17,20,21,23,27,33,34,36^, with a 0.80 to 10% higher ground contact time in the WR condition compared to the UL condition. Of those, eight studies reported the ES for ground contact time^13,16,17,20,21,23,33,36^, ranging from 0.10 to 0.73.

For subgroup analysis, ground contact time for acceleration phase (SMD 0.51 (95% CI 0.14 to 0.87), Z = 2.72 (I^2^ = 0%)), WR loads ≤ 2% (SMD 0.53 (95% CI 0.15 to 0.92), Z = 2.73 (I^2^ = 0%)), and WR for lower body (SMD 0.49 (95% CI 0.10 to 0.87), Z = 2.47 (I^2^ = 0%)) were significantly (*p* ≤ 0.12) lower in UL condition compared to WR condition.

##### Flight time WR

**Figure 4:**
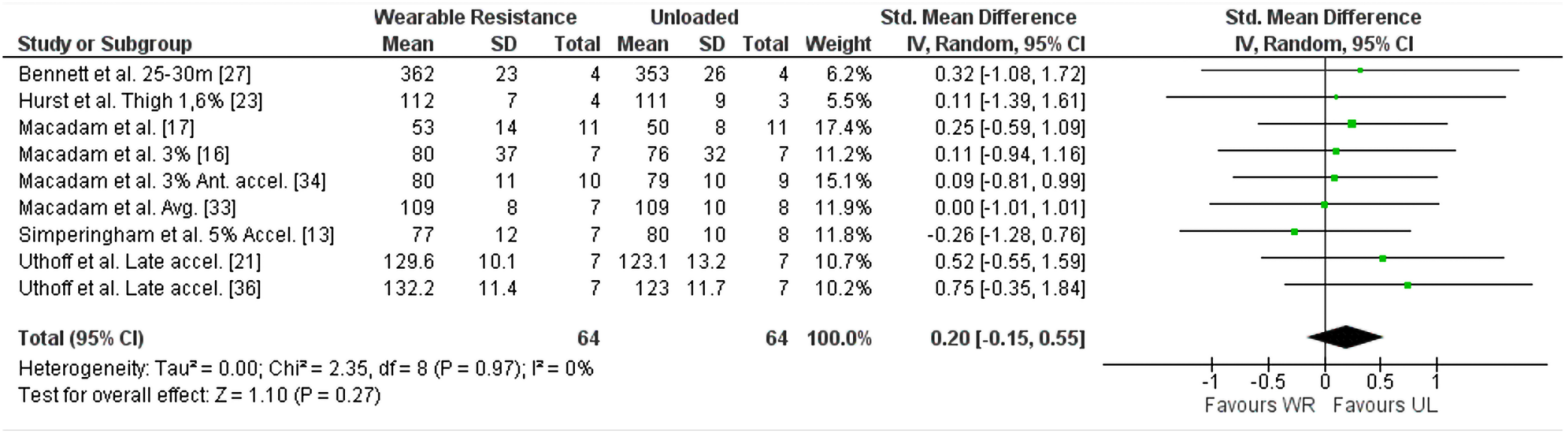
Forest plot of meta-analysis of the standardized mean difference from WR and UL conditions for flight time. accel., Acceleration phase; Ante., Anterior; CI, Confidence interval; SD, Standard deviation; UL, Unloaded condition; WR, Wearable resistance condition.

Results in meta-analysis indicated a non-significant difference in flight time between the WR condition and UL condition ([SMD] 0.20 (95% CI (− 0.15) to 0.55), Z = 1.10 (*p* = 0.27)) with a low, non-significant, average heterogeneity (I^2^ = 0% (*p* = 0.97)). The forest plot for meta-analysis is presented in Fig. 4. Nine studies reported the flight time^13,16,17,21,23,27,33,34,36^ with a percentual change ranging from − 19.35 to 7.48% in the UL condition compared to the WR condition. Of those, six studies reported the ES for flight time ^16,17,21,23,33,36^, ranging from − 0.24 to 0.79.

No sub-group analysis demonstrated significance for flight time.

##### Step Frequency WR

**Figure 5:**
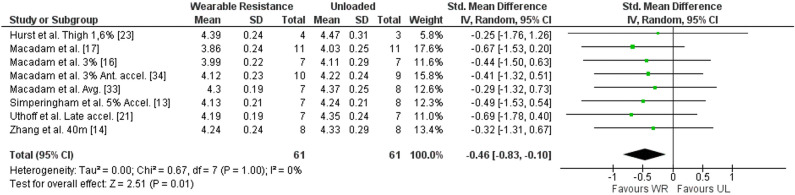
Forest plot of meta-analysis of the standardized mean difference from WR and UL conditions for step frequency. accel., Acceleration phase; Ante., Anterior; CI, Confidence interval; SD, Standard deviation; UL, Unloaded condition; WR, Wearable resistance condition.

Results in meta-analysis indicated a significantly lower step frequency for WR condition ([SMD] − 0.46 (95% CI (− 0.83) to (− 0.10)), Z = 2.51 (*p* = 0.01)) with a low, non-significant, average heterogeneity (I^2^ = 0% (*p* = 1.00)). The forest plot for meta-analysis is presented in Fig. 5. Nine studies reported step frequency ^13,14,16,17,21,23,33,34,36^ with a − 4.82 to − 0.71% lower step frequency in the WR condition compared to the UL condition. Of those, seven studies reported the ES for step frequency^13,16,17,21,23,33,36^, ranging from − 0.92 to 0.47.

For subgroup analysis, step frequency for acceleration phase (SMD − 0.45 (95% CI (− 0.89) to (− 0.02)), Z = 2.05 (I^2^ = 0%)), WR loads ≤ 2% (SMD − 0.48 (95% CI (− 0.94) to (− 0.01)), Z = 2.01 (I^2^ = 0%)), and WR for upper body (SMD − 0.68 (95% CI (− 1.35) to (− 0.00)) were significantly (*p* ≤ 0.05) lower in WR condition compared to UL condition.

##### Step length WR

**Figure 6:**
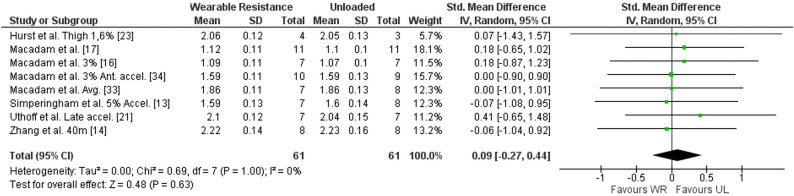
Forest plot of meta-analysis of the standardized mean difference from WR and UL conditions for step length. accel., Acceleration phase; Ante., Anterior; CI, Confidence interval; SD, Standard deviation; UL, Unloaded condition; WR, Wearable resistance condition.

Results in meta-analysis indicated a non-significant difference between the WR condition and UL condition for step length ([SMD] 0.09 (95% CI − 0.27 to 0.44), Z = 0.48 (*p* = 0.63)) with a low, non-significant, average heterogeneity (I^2^ = 0% (*p* = 1.00)). The forest plot for meta-analysis is presented in Fig. 6. Nine studies reported the step length^13,14,16,17,21,23,33,34,36^ with a percentual change ranging from − 0.62 to 3.97% in the UL condition, compared to the WR condition. Of those, six studies reported the ES for step length^17,21,23,33,34,36^, ranging from 0.02 to 1.04.

No sub-group analysis demonstrated significance for step length.

Study characteristics and subgroup analysis for WR cross-over intervention are presented in Electronic Supplementary Material (Figure S14 to S27 and Table S1, respectively).

#### Cross-over WV interventions

Five studies presented a cross-over design with a WV intervention^11,12,28,30,37^. Loads applied varied between 7 to 40% of the sample BM^11,12,28,30,37^. The most frequent load used was 20% in three studies^11,28,37^, followed by 15% in two studies^28,37^. Sprint test distance varied between 30- to 60 m^11,28,30^, with one study performing a 6-s maximum effort test^12^ and one performing a 50-m flying sprint ^37^. The most frequent sprint test used was 30- m in two studies^11,28^.

##### Sprint performance WV

**Figure 7:**
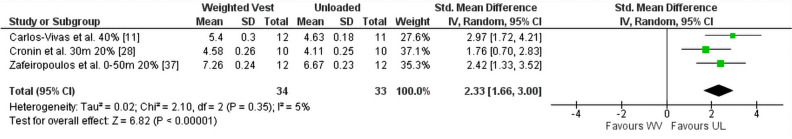
Forest plot of meta-analysis of the standardized mean difference from WV and UL conditions for sprint time. CI, Confidence interval; SD, Standard deviation; UL, Unloaded condition; WV Weighted vest condition.

Results in meta-analysis indicated a significantly lower sprint time for UL condition ([SMD] 2.33 (95% CI 1.66 to 3.00), Z = 6.82 (*p* < 0.01)) with a low, non-significant, average heterogeneity (I^2^ = 5% (*p* = 0.35)). The forest plot for meta-analysis is presented in Fig. 7. Three studies reported the sprint time^11,28,37^ with a 2.52 to 16.63% higher sprint time in the WV condition compared to the UL condition. Of those, only one study reported the ES for sprint time^11^, ranging from moderate to very large.

##### Ground contact time WV

**Figure 8:**
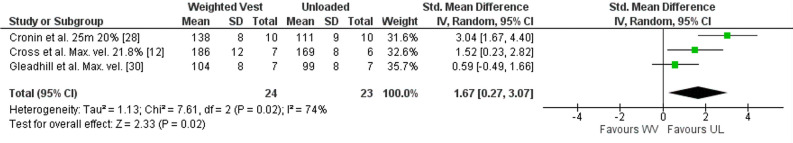
Forest plot of meta-analysis of the standardized mean difference from WV and UL conditions for ground contact time. CI, Confidence interval; Max. vel., Maximum velocity; SD, Standard deviation; UL, Unloaded condition; WV, Weighted vest condition.

Results in meta-analysis indicated a significantly lower ground contact time for UL condition ([SMD] 1.67 (95% CI 0.27 to 3.07), Z = 2.33 (*p* = 0.02)) with a high, significant, average heterogeneity (I^2^ = 74% (*p* = 0.02)). The forest plot for meta-analysis is presented in Fig. 8. Three studies reported the ground contact time^12,28,30^ with a 1.35 to 24.32% higher ground contact time in the WV condition compared to the UL condition. Of those, two studies reported the ES for ground contact time^12,30^, ranging from 0.70 to 1.71.

##### Flight time WV

**Figure 9:**
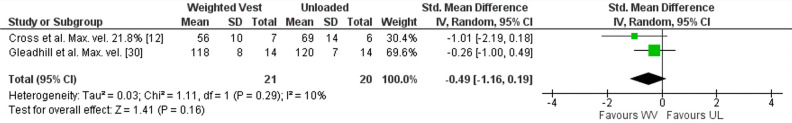
Forest plot of meta-analysis of the standardized mean difference from WV and UL conditions for flight time. CI, Confidence interval; Max. vel., Maximum velocity; SD, Standard deviation; UL, Unloaded condition; WV, Weighted vest condition.

Results in meta-analysis indicated a non-significant difference between the WV condition and UL condition for flight time ([SMD] − 0.49 (95% CI − 1.16 to 0.19), Z = 1.41 (*p* = 0.16)) with a low, non-significant, average heterogeneity (I^2^ = 10% (*p* = 0.29)). The forest plot for meta-analysis is presented in Fig. 9. Two studies reported the flight time^12,30^ with a − 1.67 to − 27% lower flight time in the WV condition compared to the UL condition. ES for flight time ^12,30^ ranged from − 0.28 to − 1.50.

##### Step frequency WV

**Figure 10:**
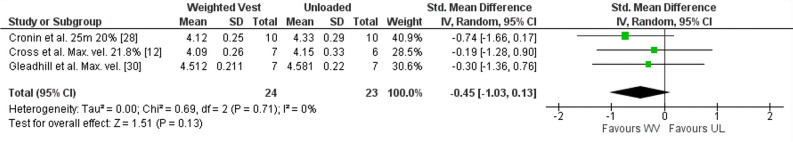
Forest plot of meta-analysis of the standardized mean difference from WV and UL conditions for step frequency. CI, Confidence interval; Max. vel., Maximum velocity; SD, Standard deviation; UL, Unloaded condition; WV, Weighted vest condition.

Results in meta-analysis indicated a non-significant difference between the WV condition and UL condition for step frequency ([SMD] − 0.45 (95% CI (− 1.03) to 0.13), Z = 1.51 (*p* = 0.13)) with a low, non-significant, average heterogeneity (I^2^ = 0% (*p* = 0.71)). The forest plot for meta-analysis is presented in Fig. 10. Three studies reported the step frequency ^12,28,30^ with a percentual change ranging from 0.72 to − 4.89% in the UL condition compared to the WV condition. Of those, only one study reported the ES for step frequency^12,30^, ranging from − 0.17 to − 0.34.

##### Step length WV

**Figure 11:**
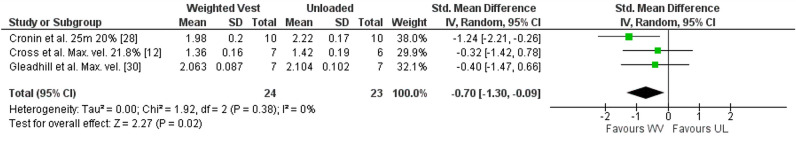
Forest plot of meta-analysis of the standardized mean difference from WV and UL conditions for FT. CI, Confidence interval; Max. vel., Maximum velocity; SD, Standard deviation; SL, Step length; UL, Unloaded condition; WV, Weighted vest condition.

Results in meta-analysis indicated a significantly lower step length for WV condition ([SMD] − 0.70 (95% CI (− 1.30) to (− 0.09)), Z = 2.27 (*p* = 0.02)) with a low, non-significant, average heterogeneity (I^2^ = 0% (*p* = 0.38)). The forest plot for meta-analysis is presented in Fig. 11. Three studies reported the step length^12,28,30^ with a − 1.95 to − 10.81% lower step length in the WV condition compared to the UL condition. Of those, two studies reported the ES for step length^12,30^, ranging from − 0.33 to − 0.55.

The Electronic Supplementary Material (Table S2) presents study characteristics for WV cross-over intervention.

#### Longitudinal WR interventions

Only one study conducted a longitudinal design with a WR intervention^15^. A 1% BM load was applied to the shank. The sprint test used for performance assessment was 30-m.

##### Sprint performance WR

Results for sprint time on pre-post intervention in the WR group ranged from 0.22 to 1.59%, and pre-post intervention in the CON group ranged from 2.20 to 7.87%. ES varied from 0.06 to 0.29 in pre-post intervention in the WR group, whereas ES varied from 0.36 to 1.25 in the CON group.

The Electronic Supplementary Material (Table S3) presents study data for WR longitudinal intervention.

#### Longitudinal WV interventions

Four studies presented a longitudinal design with a WV intervention^10,18,19,35^. Loads applied varied from 8 to 18.9% of the sample BM^10,18,19,35^. Sprint test distance varied between 10- to 54.9 m. There was no difference in frequency concerning the load and sprint test used between the studies.

##### Sprint performance WV

**Figure 12:**
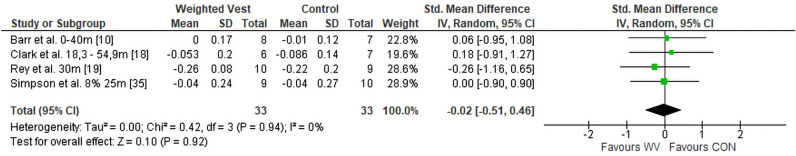
Forest plot of meta-analysis of the standardized mean difference between WV and CON intervention for sprint time. CI, Confidence interval; CON, Control group; SD, Standard deviation; WV, Weighted vest group.

Results in meta-analysis indicated no difference in sprint time between groups ([SMD] − 0.02 (95% CI (− 0.51) to 0.46), Z = 0.10 (*p* = 0.92)) with a low, non-significant, average heterogeneity (I^2^ = 0% (*p* = 0.94)). The forest plot for meta-analysis is presented in Fig. 12. All studies reported the sprint time^10,18,19,35^ with a 0 to − 9.55% change in sprint time in pre-post intervention for the WV group and a 0 to − 11.17% change in sprint time in pre-post intervention for the CON group. Three studies reported ES^10,18,19^, varying from 0 to 3.30 in pre-post intervention in the WV group, whereas ES varied from 0 to 2.50 in the CON group.

##### Ground contact time WV

**Figure 13:**
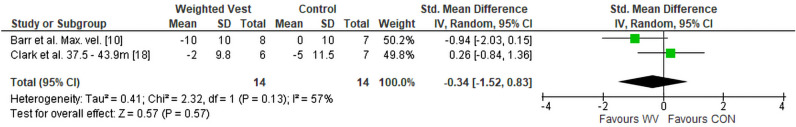
Forest plot of meta-analysis of the standardized mean difference between WV and CON intervention for ground contact time. CI, Confidence interval; CON, Control group; SD, Standard deviation; WV, Weighted vest group.

Results in meta-analysis indicated no difference in ground contact time between groups ([SMD] − 0.34 (95% CI (− 1.52) to 0.83), Z = 0.57 (*p* = 0.57)) with a high, non-significant, average heterogeneity (I^2^ = 57% (*p* = 0.13)). The forest plot for meta-analysis is presented in Fig. 13. Two studies reported the ground contact time^10,18^ with a 0 to − 9.09% change in ground contact time in pre-post intervention for the WV group and a 0 to − 3.79% change in pre-post intervention for the CON group. Two studies reported ES^10,18^ varying from 0.09 to 0.28 in pre-post intervention for the WV group, whereas ES varied from 0.1 to 0.38 in the CON group.

##### Flight time WV

**Figure 14:**
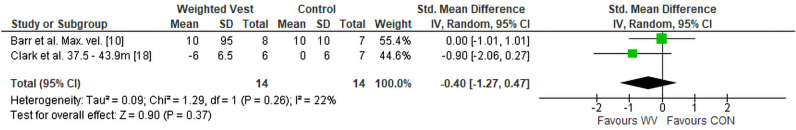
Forest plot of meta-analysis of the standardized mean difference from WV and CON intervention for flight time. CI, Confidence interval; CON, Control group; SD, Standard deviation; WV, Weighted vest group.

Results in meta-analysis indicated no difference in FT between groups ([SMD] − 0.40 (95% CI (− 1.27) to 0.47), Z = 0.90 (*p* = 0.37)) with a low, non-significant, average heterogeneity (I^2^ = 22% (*p* = 0.26)). The forest plot for meta-analysis is presented in Fig. 14. Two studies reported the flight time ^10,18^ with a − 5.40 to 9.09% change in pre-post intervention for the WV group and a 0 to 9.09% change in pre-post intervention for the CON group. Two studies reported ES^10,18^ varying from 0.25 to 1.00 in pre-post intervention in the WV group, whereas ES varied from 0 to 0.40 in the CON group.

The Electronic Supplementary Material (Table S4) presents study characteristics for WV longitudinal interventions.

## Discussion

**Figure 15:**
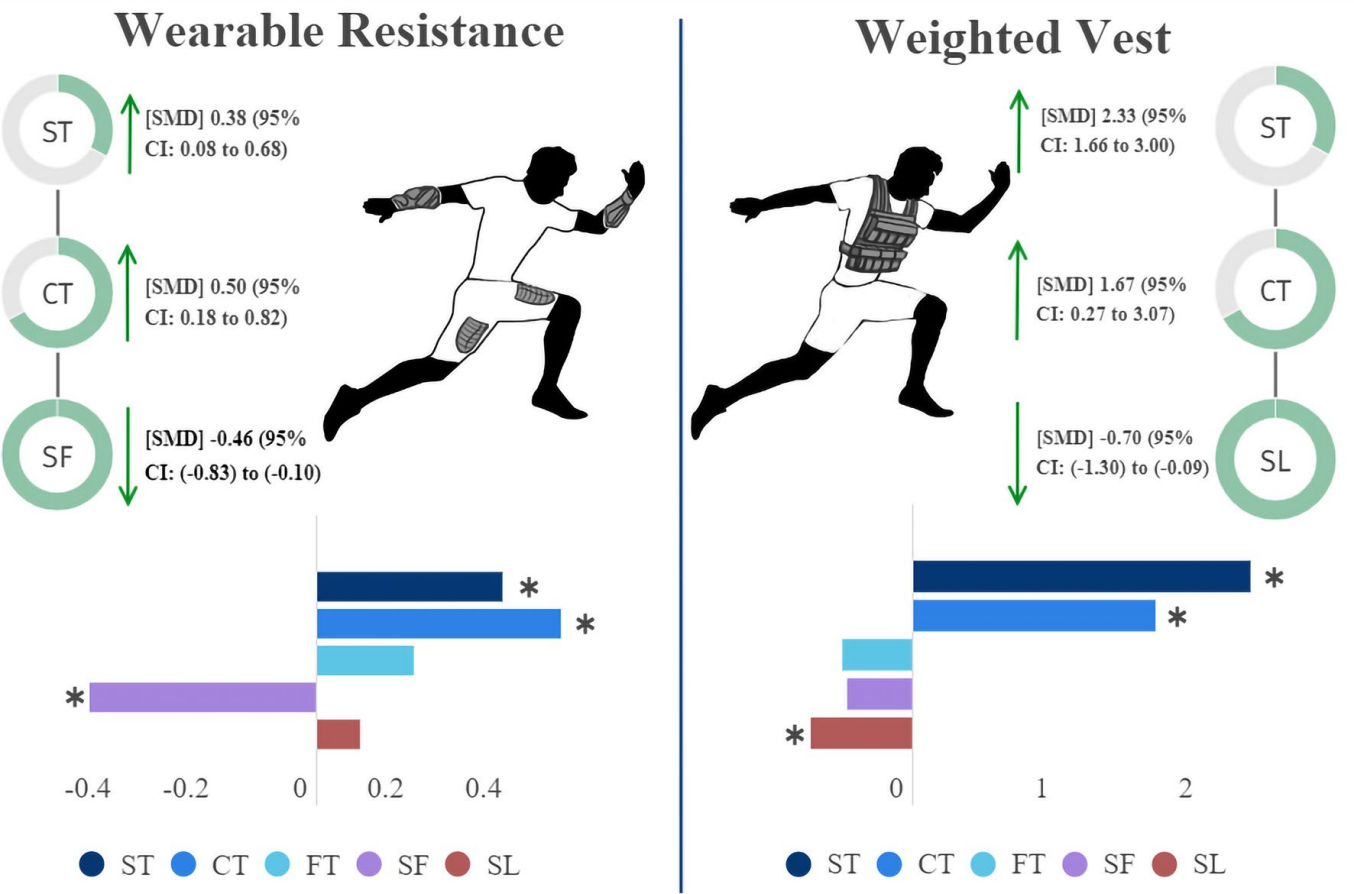
Acute kinematic change during sprint with WR/WV infographic. CT: Ground contact time; FT: Flight time; SF: Step frequency; SL: Step length; SMD: Standardize mean difference; ST: sprint time. Asterisks denote significant differences compared with the respective UL conditions.

WR and WV are loaded sprint strategies that can be implemented during training, aiming for higher performance. Due to the differences in weight and positioning used between the WV and WR, different adaptations can be found with its use. Thus, we aimed to systematically review the literature and conduct a meta-analysis on the acute and chronic kinematic and performance impact of WV and WR, comparing them. The acute findings involve: (1) WR and WV produced an increased sprint time compared to UL. Subgroup analysis also demonstrated an increase in sprint time in the acceleration sprint phase for WR condition and with WR loads ≤ 2% BM compared to UL; (2) WR and WV produced an increased ground contact time compared to UL. Subgroup analysis also demonstrated an increase in ground contact time in the acceleration sprint phase for WR condition, with WR loads ≤ 2% BM, and for WR placed in the lower body compared to UL; (3) WR produced a lower step frequency compared to UL. Subgroup analysis also demonstrated a decrease in step frequency on acceleration sprint phase for WR condition, with WR loads ≤ 2% BM, and for WR used in the upper body compared to UL; (4) WV produced a lower step length compared to UL. The main finding for longitudinal studies was: 1) The WR group maintained sprint time compared to the CON group, which decreased the sprint time. Figure 15 represents an infographic about acute kinematic changes for WR and WV.

Both WR and WV demonstrated an increase in sprint time, as shown in the meta-analysis. Sprinting with additional load can be challenging as the additional load offers resistance to the movement, decreasing the velocity^7^ and, consequently increasing the sprint time. In addition to WV and WR, other strategies to overload the sprint movement are used in the field. Alcaraz et al.^7^ found a 5 to 6% velocity decrease with a parachute and a 12 to 14% velocity decrease with sled towing. Compared to the results of this meta-analysis, the 5 to 6% performance loss in the parachute condition is close to the performance loss found in the WR condition (i.e., 0 to 7.4%). Conversely, 12 to 14% of performance lost in sled towing conditions are closer to WV condition in this meta-analysis (i.e., 2.52 to 16.63%). The loss in performance can be explained by the additional load used. In sled towing conditions, loads were approximately 16% of BM. Such load is much heavier than the loads used in WR studies (i.e., 0.55 to 5%). Probably, the parachute size used was not enough to largely reduce the performance, as seen in WV condition. On the other hand, Alcaraz et al.^7^ also investigated the effects of a weight belt (a very similar strategy to the WV) in sprinting, finding a reduction of 3% in sprint velocity. A 3% loss in performance is very similar to the lowest performance reduction found for WV in this meta-analysis (i.e., 2.52%). This reduction is reasonable as the load in the weight belt condition (9%) was close to the minimum load used in WV studies (7%). Therefore, the more load used, the more overload is promoted during sprint^7,11,39^; however, in our sub-analysis, sprinting with ≤ 2% of the BM demonstrated superiority in reducing sprint time compared to > 2% of the BM. Sprinting with WR can increase the rotational inertia of the limbs as additional resistance is added, beginning with 2.42% more inertia with 200 g and reaching 11.04% more inertia with 1000 g^9^. Thus, the higher rotational inertia from higher loads may have contributed to less impact on sprint time, ground contact time, and step length as an assistant to the swing phase during sprinting.

Regarding ground contact time, athletes have little time in contact with the ground during sprinting to generate force to move (i.e., ~ 0.1 to 0.2 s)^17,20^. Thus, the more force athletes can apply during every contact can result in better performance^40^. As a result of carrying more load due to WV or WR strategies, athletes need to spend more time during the contact to produce a similar amount of propulsive force^17,28,34^. Not only WV and WR but also the use of external load in resistive sprint techniques constantly demonstrate an increase in ground contact time^28,41^. During the acceleration phase, the ground contact time is higher, compared to the maximal velocity phase, as athletes needed more time to produce the necessary force^42^ and overcome inertia. In this context, the results from this meta-analysis confirm that the acceleration phase experienced higher ground contact time using WR and, thus, a higher sprint time. A greater horizontal force is needed in the acceleration phase compared to the maximum velocity phase to overcome inertia during sprint initiation, resulting in a higher propulsive impulse^42^, especially with additional load. Unlike ground contact time, flight time does not seem to change during an loaded sprint. An unchanged flight time seems a constant outcome even during other loaded/resistive strategies, like sled and parachute, irrespective of the load^43,44^. However, to maintain flight time constant, athletes must apply more force on the ground, as shown with increased electromyography for lower limb muscles, associated with a higher ground contact time^44^, ensuring a propulsion similar to UL condition, with little change on flight time.

The meta-analysis results demonstrated a significant decrease in step frequency for WR and step length for WV. Step frequency and step length are related and determinants of the final velocity (i.e., Velocity = step frequency × step length). Thus, if an athlete's performance is affected, one or both step kinematics will also be affected. Therefore, one reason to explain the decreased sprint time on the WR condition is the decreased step frequency, whereas the decreased sprint time on WV is due to a decreased step length. During sprinting, higher times to complete a full stride cycle (ground contact time + flight time) will negatively influence the number of steps an athlete completes (step frequency)^34,45^. This explains why step frequency decreases in the WR condition as the ground contact time increases.

Conversely, if an athlete covers short distances at each step, as seen in WV with a decreased step length, there is a necessity for a higher number of steps. Even though the higher number of steps was not large enough to demonstrate significance on step frequency for the WV condition, it affected sprint time. Elite athletes' reliance on respecting step length or step frequency is very individual^46^. Thus, we recommend step frequency-reliant athletes choose WR for their training programs (especially, but not constantly, for upper body and ≤ 2% BM WR), whereas step length-reliant chose the WV strategy.

During sprinting, leg and arm coordination is important in improving performance^47^; however, there is a slight difference between the lower and upper limbs’ contributions to propulsion during sprinting. The upward movement of the arms causes a downward force on the body that contributes to a larger force being applied to the ground and generating a larger impulse^48^. Using WR in forearms can overload arm swing, generating more vertical impulse^21^. Thus, although arms play an important role in maintaining balance, they also contribute to lower limbs’ sprint performance^49^, especially in the start and acceleration phases, where the body presents a more leaning position^47^. Sub-group analysis demonstrates a significantly increased ground contact time for WR's lower body and decreased step frequency for WR's upper body conditions. Couture et al.^50^ found an increased ground contact time with upper and lower body WR (except for 1% lower body WR), yet with no significance compared to UL. However, the authors found that during the lower body condition, the horizontal propulsive impulse increased twofold compared to the upper body condition, although both conditions demonstrated, in part, increased propulsive force compared to the UL condition. Thus, the more propulsive force needs higher ground contact time, explaining why lower body WR increased the contact time. Since more ground contact time is demanded from lower body WR, the authors expected that the step frequency would also present a significant decrease in the WR in the lower limbs, which did not occur. Only upper-body WR showed a lower step frequency. We believe that the greater presence of studies that evaluated the step frequency in the maximum velocity phases in sub-group analysis for the lower body compared to the sub-analysis for the upper body may have masked the effects since the acceleration phase demands more ground contact time.

The kinematic changes in acute interventions using WV and WR raise questions about long-term adaptations. Meta-analysis results indicate no difference between CON ground and WV group, however, with WR presenting a beneficial effect in maintaining a better sprint time, compared to the decreased sprint time in the CON group. The studies included in this meta-analysis with long-term use of WV demonstrated significant improvement in sprint performance after a training period, regardless of the condition (loaded or unloaded sprint)^18,19^. High-intensity sprint training positively influences muscle fiber type shift, enhances stretch reflex (increasing force production), and increases the capability to produce maximal forces^51–53^, thus increasing performance. Therefore, positive muscle adaptations are not necessarily greater using WV, considering sprint training.

On the other hand, in the only study analyzing long-term WR training, authors found a better result in performance with WR when compared to CON^15^. However, even in the WR group, there was no pre- and post-performance enhancement. What happened is that the WR group lost less performance during the study period than the CON group, showing a significant difference between the groups post-training. Still, only one study investigated WR. Therefore, the additional load does not bring benefits beyond the training stimulus itself.

Initially, a question was raised about the efficiency of the vertical overload applied by WV and WR, which is different from horizontal overload from sled towing, for example. However, Alcaraz et al.^54^ drew similar conclusions with sled towing training to improve performance. Nonetheless, Alcaraz et al.^54^ sub-group analysis demonstrated a better ES for training periods longer than six weeks. All studies included in this meta-analysis used 6 weeks or less of training period, except for Clark et al.^18^, which used seven. Thus, longer training periods may result in larger benefits from resisted/loaded sprint training. About the load applied, Alcaraz et al.^54^ observed a higher effect size and significant improvements with loads lower than 20%. The better results found by Alcaraz et al.^54^ with lower load can probably be explained by the loss of sprint technique when higher loads are applied. Some studies, like Clark et al.^18^, used a 10% velocity loss as a determinant to establish the load magnitude. Thus, it is important to control the load magnitude, especially when higher loads are used, to not disturb the athlete's technique excessively.

This meta-analysis demonstrated that long-term WV training did not show any statistical difference from CON in reducing or enhancing ground contact time or flight time. Literature shows that the fastest athletes demonstrate decreased ground contact time compared to their slower counterparts^55^. However, even if studies support that long-term sprint training affects the contact time, it appears to be non-dependent upon overloading techniques used in sprint training^18^. A more efficient force production (and then less contact time) results from sprint training itself. The same partially occurs with Alcaraz et al.^43^, where athletes in UL group significantly reduce the ground contact time in the maximal velocity phase. However, the sled towing group seems to increase the ground contact time, although it is not significant. Like the contact time, flight time presents minimal changes during UL sprint training. Accordingly, Clark et al.^18^ demonstrated a moderate decrease in flight time only in WV condition, compared to UL and sled towing, however, with no significant difference. Thus, using WV does not affect long-term ground contact time and flight time, presenting small effects in the sprint technique.

## Future perspectives

The acute use of WV and, especially, WR, has been frequently investigated. The main concern about WV and WR is in longitudinal studies. Even if acute interventions demonstrated alterations in kinematic parameters, the low set of evidence until now did not support a higher effectiveness of WV or WR training. More studies are needed to investigate the longitudinal effects of WV and WR, especially above six weeks, and compare different load magnitudes. Specifically, about WR, there are no studies regarding kinematic changes with long-term training.

## Limitations

The major limitation of this meta-analysis is the lack of information about longitudinal studies. Care should be taken when interpreting longitudinal conclusions on the use of WR and WV. Yet, only a few studies were considered high-quality.

## Conclusions

Using WV and WR in field sports demonstrates overload sprint gestures acutely. Compared to UL conditions, higher ground contact time and sprint time are found with the use of WR and WV during sprinting. However, the use of WR presents lower step frequency, which is more suitable for step frequency-reliant athletes. Conversely, WV presents lower step length, being more suitable for step length-reliant athletes. Although promising for chronic performance improvement, coaches and athletes should carefully consider WV and WR use since there is no supporting evidence that WV or WR will impact sprint performance, ground contact time, and flight times.

### Supplementary Information

Supplementary Information.

## Data Availability

The datasets used and/or analyzed during the current study available from the corresponding author on reasonable request.
