# Supplementary material for: The use of wearable resistance and weighted vest for sprint performance and kinematics: a systematic review and meta-analysis
Source: Sci Rep. 2024 Mar 5;14:5453. doi: 10.1038/s41598-024-54282-8 (PMC10915135; doi:10.1038/s41598-024-54282-8)
Supplement: Supplementary file 1 — Supplementary Information. [file 41598_2024_54282_MOESM1_ESM.docx]

**The use of wearable resistance and weighted vest for sprint performance and kinematics: A Systematic Review and Meta-Analysis.**


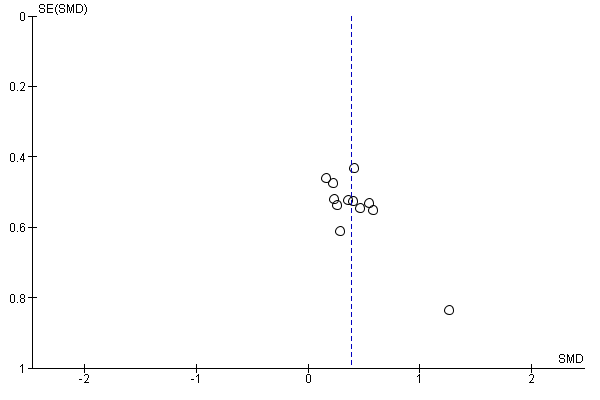


Eletronic Supplementary Material Figure S1 - Funnel plot of comparison outcome: Sprint Time for cross-over studies with Wearable Resistance.

Abbreviations: SE, Standard error; SMD Mean difference.


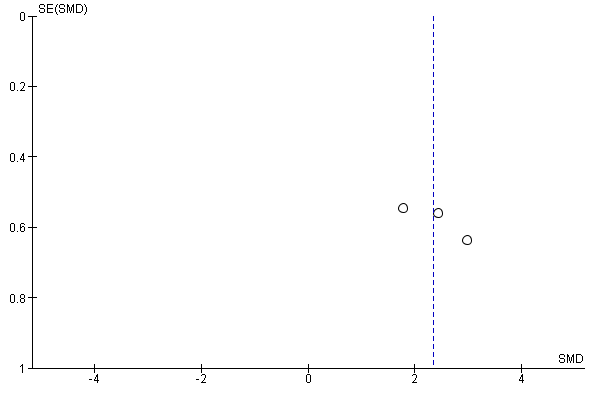


Eletronic Supplementary Material Figure S2 - Funnel plot of comparison outcome: Sprint Time for Cross-over studies with Weighted vest.

Abbreviations: SE, Standard error; SMD Mean difference.


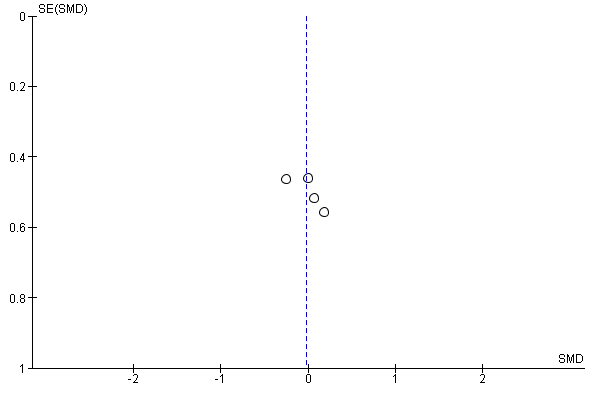


Eletronic Supplementary Material Figure S3 - Funnel plot of comparison outcome: Sprint Time for Longitudinal studies with Weighted Vest.

Abbreviations: SE, Standard error; SMD Mean difference.


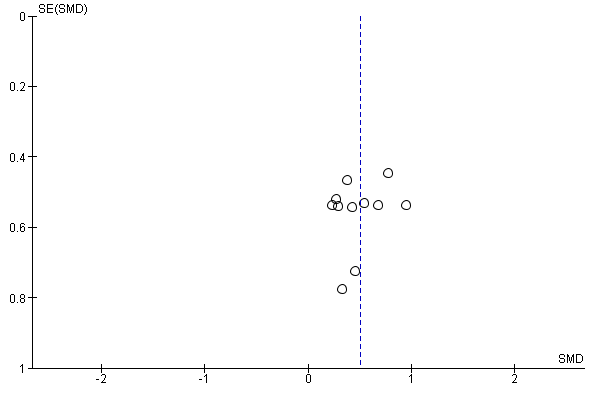


Eletronic Supplementary Material Figure S4 - Funnel plot of comparison outcome: Ground contact time for Cross-over studies with Wearable Resistance.

Abbreviations: SE, Standard error; SMD Mean difference.


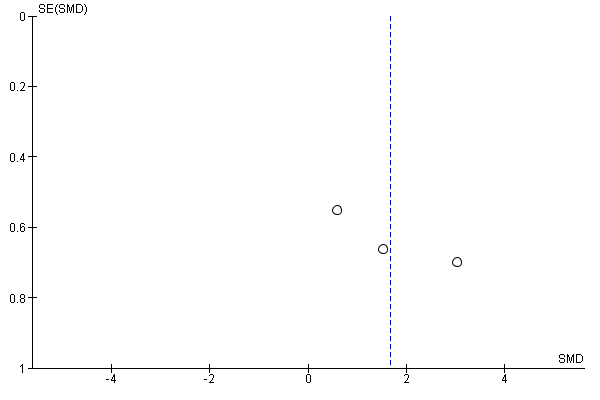


Eletronic Supplementary Material Figure S5 - Funnel plot of comparison outcome: Ground contact time for Cross-over studies with Weighted Vest.

Abbreviations: SE, Standard error; SMD Mean difference.


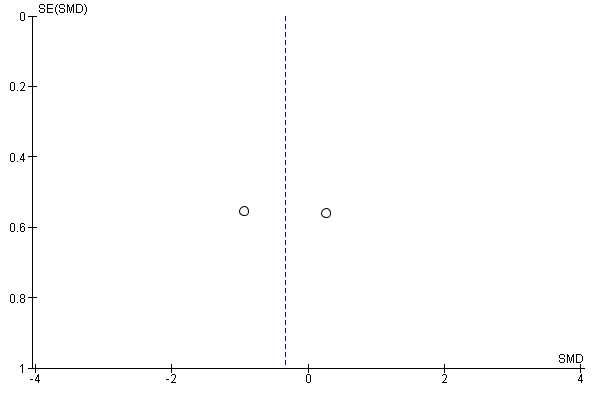


Eletronic Supplementary Material Figure S6 - Funnel plot of comparison outcome: Ground contact time for Longitudinal studies with Weighted Vest.

Abbreviations: SE, Standard error; SMD Mean difference.


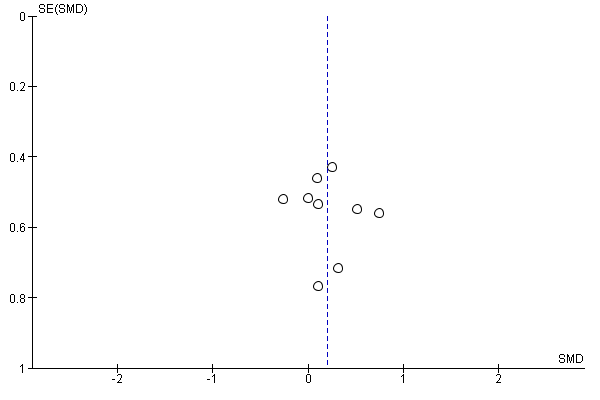


Eletronic Supplementary Material Figure S7 - Funnel plot of comparison outcome: Flight time for Cross-over studies with Wearable Resistance.

Abbreviations: SE, Standard error; SMD Mean difference.


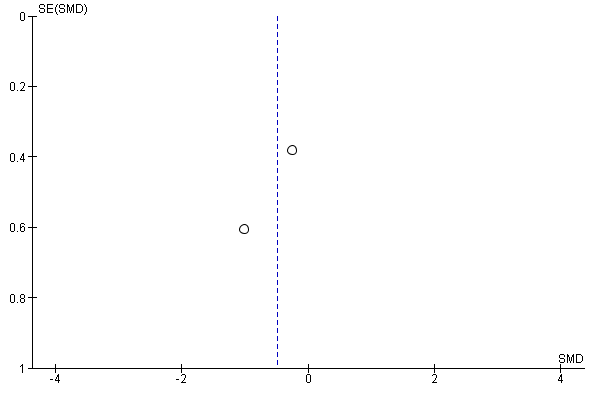


Eletronic Supplementary Material Figure S8 - Funnel plot of comparison outcome: Flight time for Cross-over studies with Weighted Vest.

Abbreviations: SE, Standard error; SMD Mean difference.


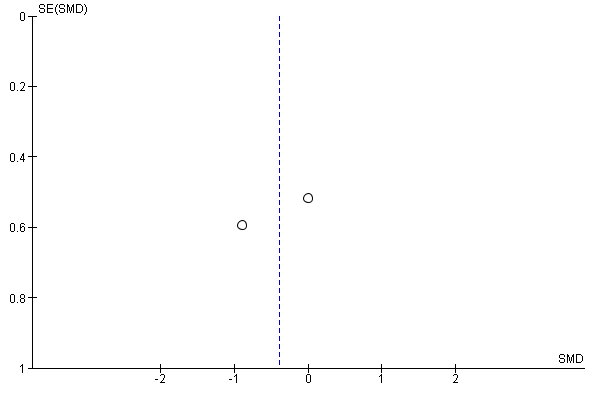


Eletronic Supplementary Material Figure S9 - Funnel plot of comparison outcome: Flight time for Longitudinal studies with Weighted Vest.

Abbreviations: SE, Standard error; SMD Mean difference.


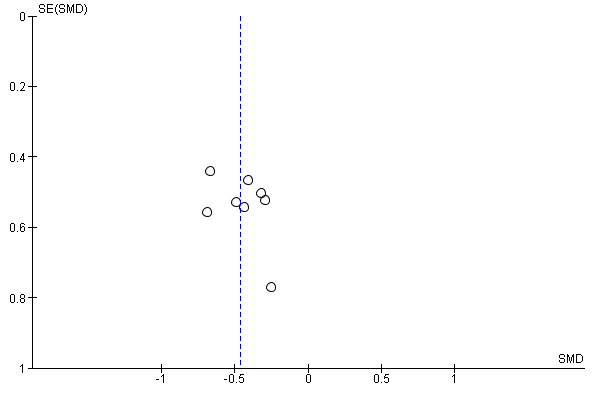


Eletronic Supplementary Material Figure S10 - Funnel plot of comparison outcome: Step Frequency for Cross-over studies with Wearable Resistance.

Abbreviations: SE, Standard error; SMD Mean difference.


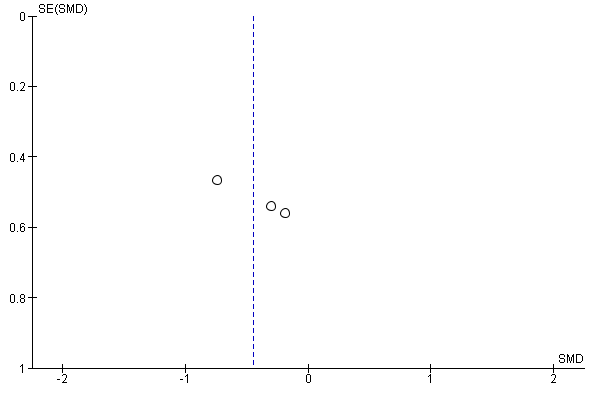


Eletronic Supplementary Material Figure S11 - Funnel plot of comparison outcome: Step Frequency for Cross-over studies with Weighted Vest.

Abbreviations: SE, Standard error; SMD Mean difference.


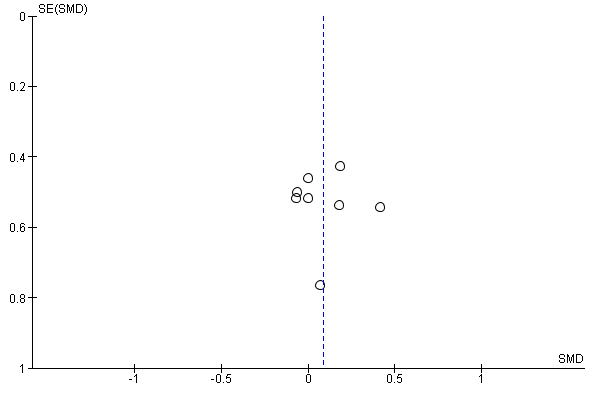


Eletronic Supplementary Material Figure S12 - Funnel plot of comparison outcome: Step length for Cross-over studies with Wearable Resistance.

Abbreviations: SE, Standard error; SMD Mean difference.


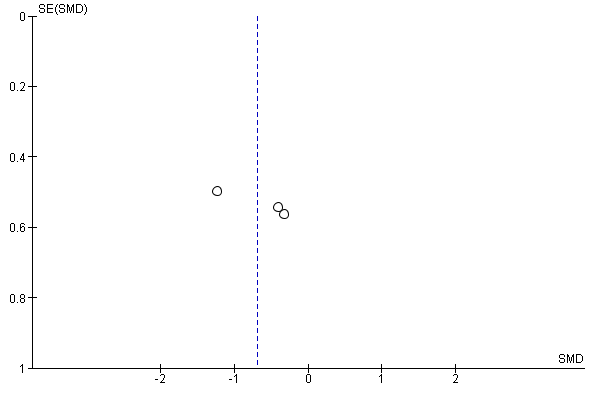


Eletronic Supplementary Material Figure S13 - Funnel plot of comparison outcome: Step length for Cross-over studies with Weighted Vest.

Abbreviations: SE, Standard error; SMD Mean difference.


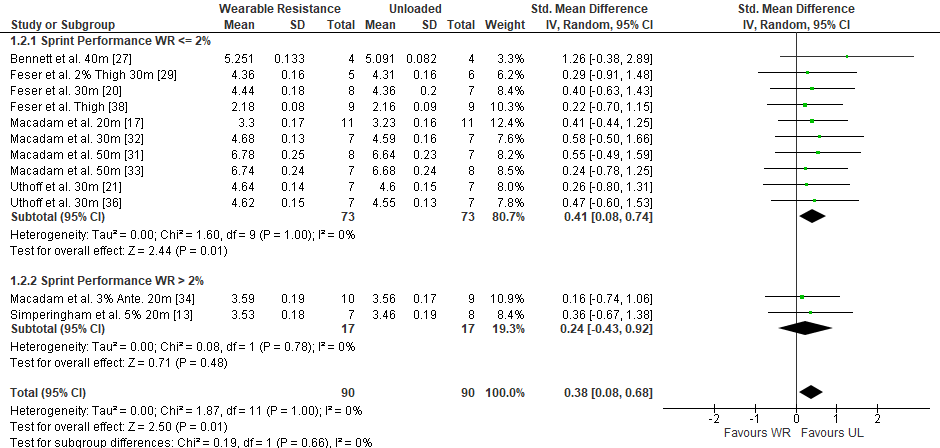


Eletronic Supplementary Material Figure S14 - Forest plot for cross-over studies on sprint performance using wearable resistance: Sub-analysis for load. Abbreviations: CI, Confidence Interval; UL, Unloaded; WR, Wearable Resistance.


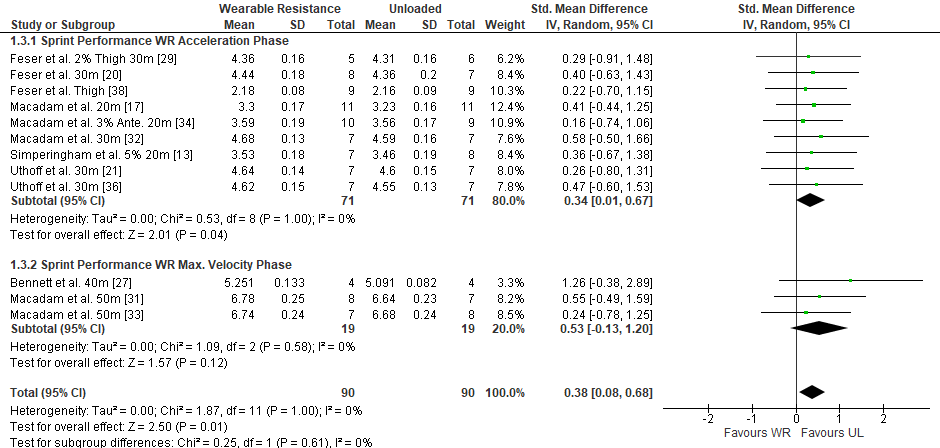


Eletronic Supplementary Material Figure S15 - Forest plot for cross-over studies on sprint performance using wearable resistance: Sub-analysis for sprint phase. Abbreviations: CI, Confidence Interval; UL, Unloaded; WR, Wearable Resistance.


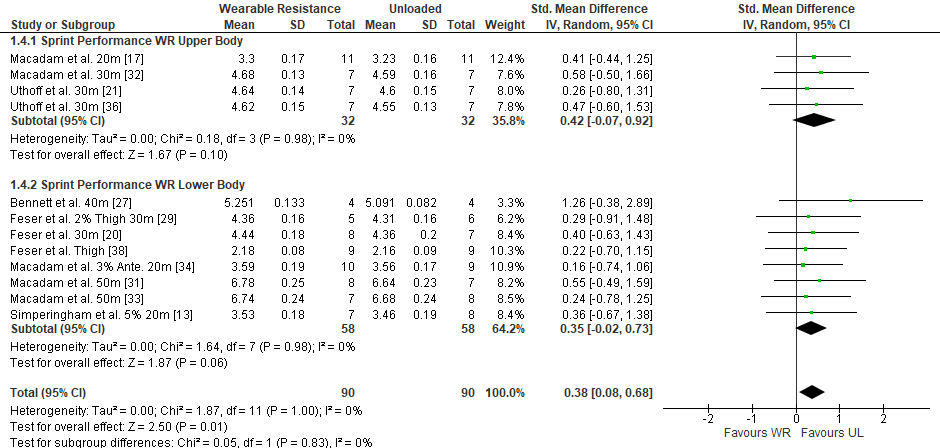


Eletronic Supplementary Material Figure S16 - Forest plot for cross-over studies on sprint performance using wearable resistance: Sub-analysis for wearable resistance positioning. Abbreviations: CI, Confidence Interval; UL, Unloaded; WR, Wearable Resistance.


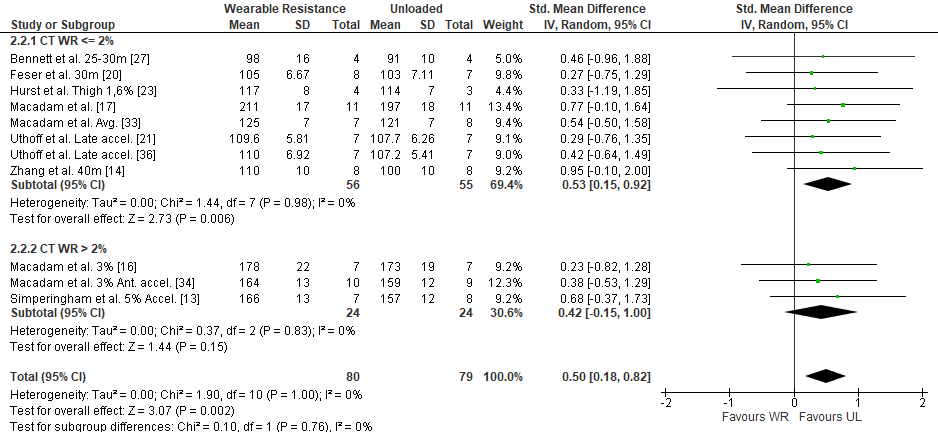


Eletronic Supplementary Material Figure S17 - Forest plot for cross-over studies on ground contact time using wearable resistance: Sub-analysis for load. Abbreviations: CI, Confidence Interval; CT, Ground Contact Time; UL, Unloaded; WR, Wearable Resistance.


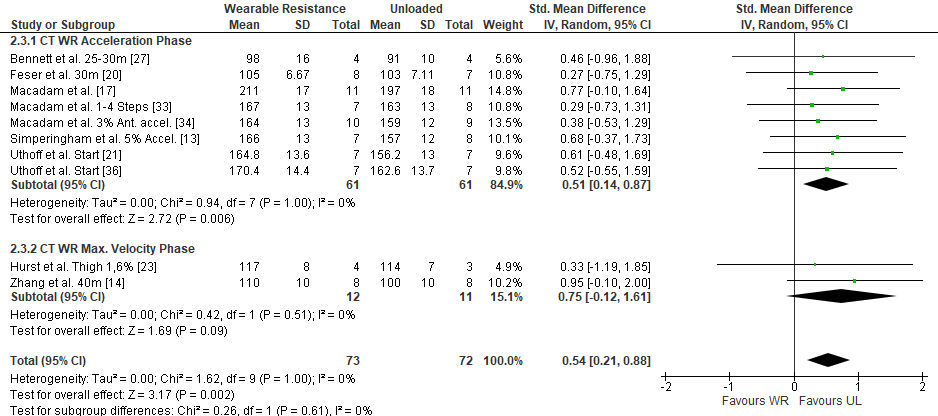


Eletronic Supplementary Material Figure S18 - Forest plot for cross-over studies on ground contact time using wearable resistance: Sub-analysis for sprint phase. Abbreviations: CI, Confidence Interval; CT, Ground Contact Time; UL, Unloaded; WR, Wearable Resistance.


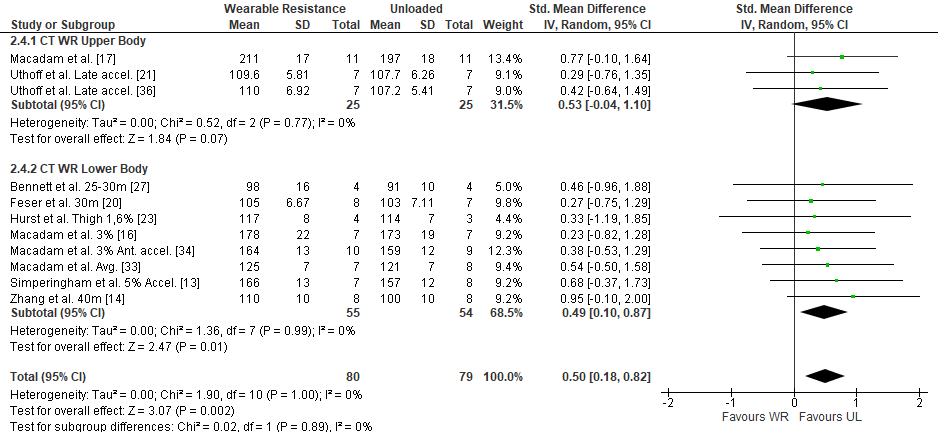


Eletronic Supplementary Material Figure S19 - Forest plot for cross-over studies on ground contact time using wearable resistance: Sub-analysis for wearable resistance positioning. Abbreviations: CI, Confidence Interval; CT, Ground Contact Time; UL, Unloaded; WR, Wearable Resistance.


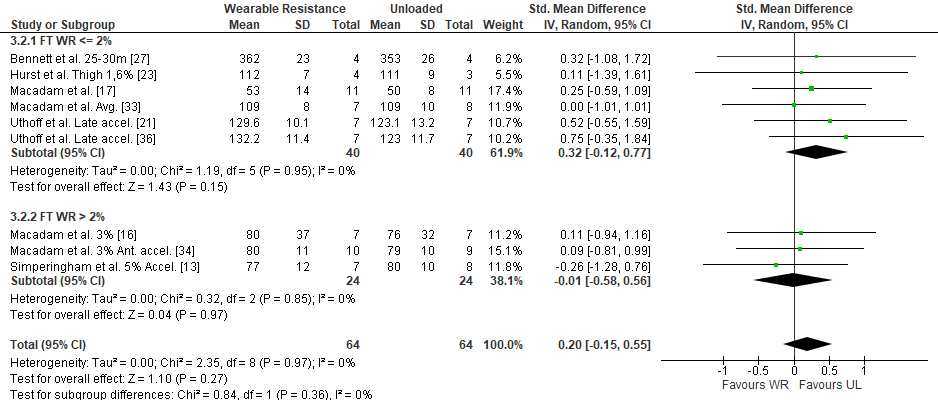


Eletronic Supplementary Material Figure S20 - Forest plot for cross-over studies on flight time using wearable resistance: Sub-analysis for load. Abbreviations: CI, Confidence Interval; FT, Flight Time; UL, Unloaded; WR, Wearable Resistance.


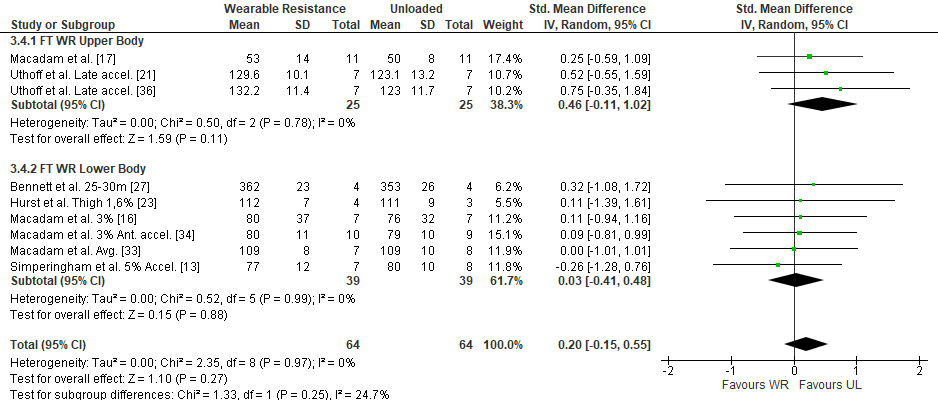


Eletronic Supplementary Material Figure S21 - Forest plot for cross-over studies on flight time using wearable resistance: Sub-analysis for wearable resistance positioning. Abbreviations: CI, Confidence Interval; FT, Flight Time; UL, Unloaded; WR, Wearable Resistance.


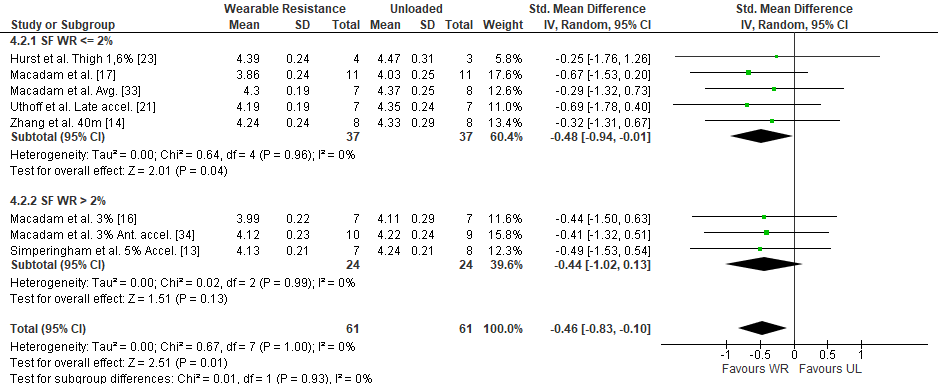


Eletronic Supplementary Material Figure S22 - Forest plot for cross-over studies on step frequency using wearable resistance: Sub-analysis for load. Abbreviations: CI, Confidence Interval; SF, Step Frequency; UL, Unloaded; WR, Wearable Resistance.


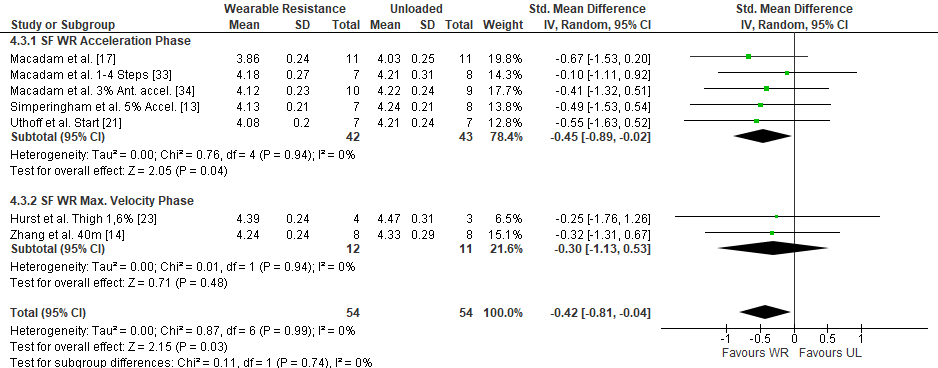


Eletronic Supplementary Material Figure S23 - Forest plot for cross-over studies on step frequency using wearable resistance: Sub-analysis for sprint phase. Abbreviations: CI, Confidence Interval; SF, Step Frequency; UL, Unloaded; WR, Wearable Resistance.


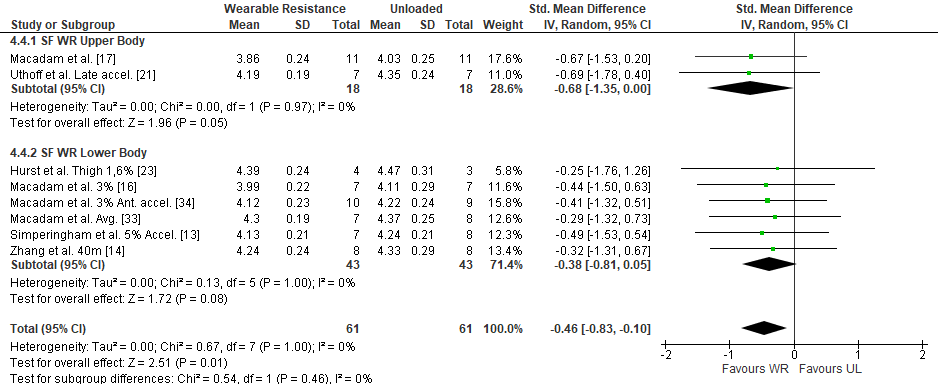


Eletronic Supplementary Material Figure S24 - Forest plot for cross-over studies on step frequency using wearable resistance: Sub-analysis for wearable resistances positioning. Abbreviations: CI, Confidence Interval; SF, Step Frequency; UL, Unloaded; WR, Wearable Resistance.


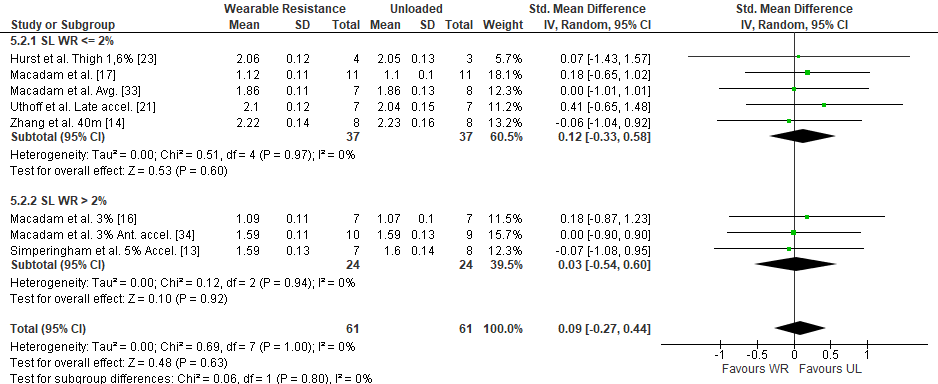


Eletronic Supplementary Material Figure S25 - Forest plot for cross-over studies on step length using wearable resistance: Sub-analysis for load. Abbreviations: CI, Confidence Interval; SL, Step Length; UL, Unloaded; WR, Wearable Resistance.


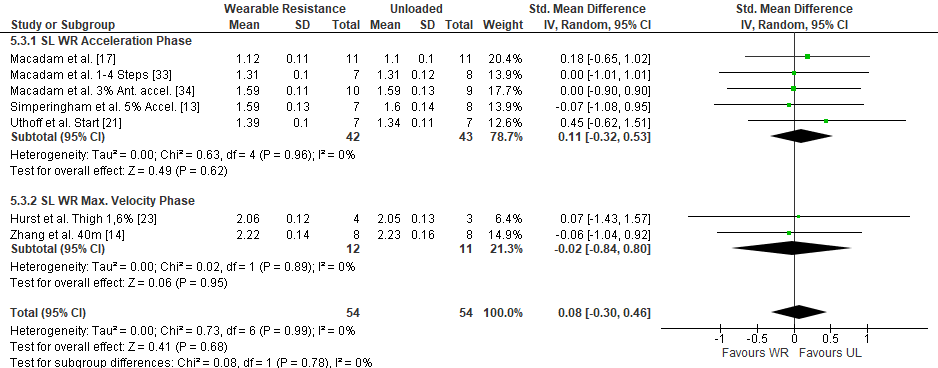


Eletronic Supplementary Material Figure S26 - Forest plot for cross-over studies on step length using wearable resistance: Sub-analysis for sprint phase. Abbreviations: CI, Confidence Interval; SL, Step Length; UL, Unloaded; WR, Wearable Resistance.


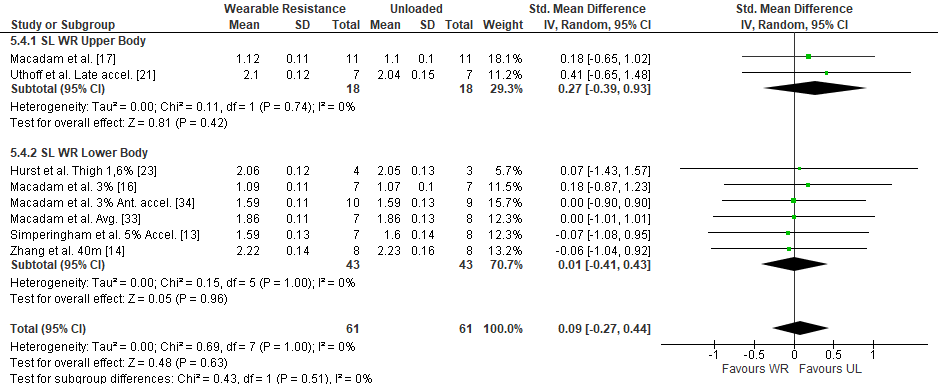


Eletronic Supplementary Material Figure S27 - Forest plot for cross-over studies on step length using wearable resistance: Sub-analysis for wearable resistance positioning. Abbreviations: CI, Confidence Interval; SL, Step Length; UL, Unloaded; WR, Wearable Resistance.

Eletronic Supplementary Material Table S1 – Study characteristics for WR cross-over interventions.

| Study | Intervention | Study objective | Sprint Test | WR/WV Load (%BM) | WR/WV Placement | WR/WV Sprint Performance (velocity and time)/Kinematic Results | WR/WV Sprint Kinematic Results (%/p/ES) | WR/WV Sprint Performance  (%/p/ES) |
| --- | --- | --- | --- | --- | --- | --- | --- | --- |
| Bennett et al. ^27^ | Athletes completed sprints under UL condition and WR condition with performance and kinematic analysis. | To examine if lower body WR training can enhance athletes’ overall sprint performance | 40-m | 10% of the thigh and shank mass (~1.5% of the sample BM) | Thigh: anterio-posterly; shank: medio-laterally. | ↑ 40-m, 10- to 20-m, 30- to 40-m in WR compared to normal condition. | CT_(UL x WR)_: 7.69%/ >0.05/NA  FT_(UL x WR)_: 2.55% />0.05/NA | ST_0-10m (UL x WR)_: -0.58%/>0.05/NA  ST_10-20m (UL x WR)_: 4.2%/≤0.02*/NA  ST_20-30m (UL x WR)_: 3.67% />0.05/NA  ST_30-40m (UL x WR)_: 7.4%/≤0.02*/NA  ST_40-m (UL x WR)_: 3.14%/≤0.02*/NA |
| Feser et al. ^20^ | Athletes completed 2 sprints under UL and 2 sprints under WR condition with performance and kinematic analysis. | To compare 2% shank WR with UL condition in sprinting contact times and force waveforms. | 50-m | 2% | WR attached to the shank aligned with the long axis of the segment. | ↑ CT at 30-m with WR. | CT_(UL x 5-m Shank)_: -1.40%/0.18/0.15  CT_(UL x 10-m Shank)_: 0.80%/0.42/0.12  CT_(UL x 20-m Shank)_: 0.90%/0.15/0.13  CT_(UL x 30-m Shank)_: 1.94%/0.03*/0.25 | ST_(UL x 5-m Shank)_: 0.77%/NA/NA  ST_(UL x 10-m Shank)_: 1.51%/NA/NA  ST_(UL x 20-m Shank)_: 1.87%/NA/NA  ST_(UL x 30-m Shank)_: 1.83%/NA/NA |
| Feser et al. ^29^ | Athletes completed 2 testing days. Each day, athletes completed 2 sprints under WR thigh or shank and 2 sprints under UL. | To determine the effect of WR attached to the shank or thigh on horizontal F-v and impulse measures during sprint running acceleration. | 50-m | 2% | Attached to the shank (in line with the long axis of the segment, equally distributed) or thigh (2/3 anteriorly and 1/3 posteriorly horizontally). | ↑ WR thigh ST in 10-/20-/30-m; ↑ WR shank in all sprints time | NA | ST_5-m (Thigh x Shank)_: 1.56%/0.06/0.40  ST_5-m (UL x Thigh)_: 0%/0.07/ 0.00  ST_5-m (UL x Shank)_: 1.56%/<0.01*/0.44  ST_10-m (Thigh x Shank)_: 1%/0.03*/0.29  ST_10-m (UL x Thigh)_: 1.01%/0.02*/ 0.29  ST_10-m (UL x Shank)_: 2.02%/<0.01*/0.57  ST_20-m (Thigh x Shank)_: 0.93%/0.04*/0.25  ST_20-m (UL x Thigh)_: 0.94%/0.01*/ 0.26  ST_20-m (UL x Shank)_: 1.88%/<0.01*/0.52  ST_30-m (Thigh x Shank)_: 0.92%/0.04*/0.24  ST_30-m (UL x Thigh)_: 1.16%/<0.01*/ 0.31  ST_30-m (UL x Shank)_: 2.09%/<0.01*/0.55 |
| Feser ^38^ | Athletes completed 2 sprints under 3 different conditions (WR shank/ WR thigh/ UL) | To quantify the acute effects of WR shank and WR thigh on kinematics during acceleration sprint phase | 10-m | 2% | WR thigh was positioned distally in a horizontal orientation (2/3 anteriorly and 1/3 posteriorly)/ WR shank was positioned along the long axis of the shank balanced around the limb. | ↑ WR shank ST compared to UL | NA | ST_10-m (UL x Thigh)_: 0.92%/0.13/0.24  ST_10-m (UL x Shank)_: 1.39%/0.02*/0/33 |
| Hurst et al. ^23^ | Athletes completed 2 sprint tests under 3 different conditions (WR shank/ WR thigh/ UL). | To quantify the acute effects of light wearable loads attached to shank or thigh during sprinting on kinematics and kinetics. | 40-m | 0.56% WR shank /1.68% WR thigh | WR were attached to the shank (positioned at 62 ± 11% of the distance from the proximal end of the segment, anteriorly)/ WR attached to the thigh (positioned at 76 ± 4% of the distance from the proximal end of the segment, anteriorly) | ↑ CT in both WR conditions | CT_(Thigh x Shank)_: - 1.71%/NA/NA  CT_(UL x Shank)_: 0.88%/>0.05/0.2  CT_(UL x Thigh)_: 2.63%/<0.05*/0.35  FT_(Thigh x Shank)_: 0.89%/NA/NA  FT_(UL x Shank)_: 1.8%/>0.05/0.21  FT_(UL x Thigh)_: 0.9%/>0.05/0.06  SL_(Thigh x Shank)_: 0%/NA/NA  SL_(UL x Shank)_: 0.49%/>0.05/0.07  SL_(UL x Thigh)_: 0.49%/>0.05/0.04  SF_(Thigh x Shank)_: 0%/NA/NA  SF_(UL x Shank)_: -1.79%/>0.05/-0.23  SF_(UL x Thigh)_: -1.79%/>0.05/-0.24 | NA |
| Macadam et al. ^31^ | Athletes completed 2 sprint tests under 2 different conditions (WR/UL). | To understand the effects of WR on angular work of the thigh during sprinting. | 50-m | 2% | Placed in the thigh with 2/3 of the load anteriorly and 1/3 posteriorly. | ↑ 50-m in WR condition. | NA | ST_10-m (UL x WR)_: 1.39%/>0.05/0.38  ST_50-m (UL x WR)_: 2.11%/<0.05*/0.55 |
| Macadam et al. ^32^ | Athletes completed 2 sprint tests under WR forearm condition and 1 sprint test under UL condition. | To examine the acute effects of forearm WR on the sprinting F-V profile. | 30-m | 2% | Evenly placed around the forearms | ↑ 10-/20-/30-m ST in WR condition. | NA | ST_5-m (UL x WR)_: 3.31%/0.076/0.46  ST_10-m (UL x WR)_: 2.69%/0.029*/0.54  ST_20-m (UL x WR)_: 2.32%/0.010*/0.64  ST_30-m (UL x WR)_: 1.96%/0.031*/0.62 |
| Macadam et al. ^33^ | Athletes completed 2 sprints tests under 2 different conditions (WR/UL) | To determine the acute changes in spatio-temporal, impulse and vertical stiffness variables with WR over ground maximal effort sprint-running | 50-m | 2% | Placed in the distal position of each thigh with 2/3 of the load anteriorly and 1/3 posteriorly. | No significant differences were noted in kinematic and performance variables between WR and UL. | CT_(Av. UL x WR)_: 3.30%/>0.05/0.51  CT_(1-4 steps UL x WR)_: 2.45%/>0.05/0.31  CT_(5-14 steps UL x WR)_: 2.52%/>0.05/0.57  CT_(15-23 steps UL x WR)_: 2.86%/>0.05/0.46  FT_(Av. UL x WR)_: 0%/>0.05/0.04  FT_(1-4 steps UL x WR)_: -3.90%/>0.05/-0.24  FT_(5-14 steps UL x WR)_: 0.92%/>0.05/0.09  FT_(15-23 steps UL x WR)_: 0.81%/>0.05/0.10  SL_(Av. UL x WR)_: 0%/>0.05/0.02  SL_(1-4 steps UL x WR)_: 0%/>0.05/0.04  SL_(5-14 steps UL x WR)_: 0.54%/>0.05/0.07  SL_(15-23 steps UL x WR)_: -0.48%/>0.05/0.06  SF_(Av. UL x WR)_: -1.60%/>0.05/-0.34  SF_(1-4 steps UL x WR)_: -0.71%/>0.05/-0.12  SF_(5-14 steps UL x WR)_: -2.49%/>0.05/-0.53  SF_(15-23 steps UL x WR)_: -1.81%/>0.05/-0.36 | ST_10-m (UL x WR)_: 0.93%/>0.05/0.31  ST_50-m (UL x WR)_: 0.90%/>0.05/0.44 |
| Macadam et al. ^16^ | Athletes completed 2 sprints tests under 4 different conditions (WR 1%/WR 2%/WR 3%/UL) | To investigate the effects of different WR load attached to the thighs on kinematics and kinetics during sprint running | Maximal effort 10-second sprint | 1%/2%/3% | Placed in the distal position of each thigh with 2/3 of the load anteriorly and 1/3 posteriorly. | ↓ SF with 2 and 3% WR load. | CT_(UL x WR 1%)_: 1.16%/>0.05/0.10-0.25  CT_(UL x WR 2%)_: 2.89%/>0.05/0.10-0.25  CT_(UL x WR 3%)_: 2.89%/>0.05/0.10-0.25  CT_(WR 1% x WR 2%)_: 1.71%/>0.05/0.10-0.25  CT_(WR 1% x WR 3%)_: 1.71%/>0.05/0.10-0.25  CT_(WR 2% x WR 3%)_: 0%/>0.05/0.10-0.25  FT_(UL x WR 1%)_: 5.26%/>0.05/0.08-0.13  FT_(UL x WR 2%)_: 2.63%/>0.05/0.08-0.13  FT_(UL x WR 3%)_: 5.26%/>0.05/0.08-0.13  FT_(WR 1% x WR 2%)_: -2.5%/>0.05/0.10-0.25  FT_(WR 1% x WR 3%)_: 0%/>0.05/0.10-0.25  FT_(WR 2% x WR 3%)_: 2.56%/>0.05/0.10-0.25  SL_(UL x WR 1%)_: 1.87%/>0.05/0.19-0.30  SL_(UL x WR 2%)_: 2.80%/>0.05/0.19-0.30  SL_(UL x WR 3%)_: 1.87%/>0.05/0.19-0.30  SL_(WR 1% x WR 2%)_: 0.92%/>0.05/0.19-0.30  SL_(WR 1% x WR 3%)_: 0%/>0.05/0.19-0.30  SL_(WR 2% x WR 3%)_: -0.91%/>0.05/0.19-0.30  SF_(UL x WR 1%)_: -1.22%/>0.05/0.19-0.47  SF_(UL x WR 2%)_: -1.95%/<0.05*/0.19-0.47  SF_(UL x WR 3%)_: -2.92%/<0.05*/0.19-0.47  SF_(WR 1% x WR 2%)_: -0.74%/>0.05/0.19-0.47  SF_(WR 1% x WR 3%)_: -1.72%/>0.05/0.19-0.47  SF_(WR 2% x WR 3%)_: -1%/>0.05/0.19-0.47 | NA |
| Macadam et al. ^34^ | Athletes performed 2 sprints running under 3 different loading condition (3% AWR; 3% PWR; UL). | To determine the acute changes of 3% WR attached to the lower limb (anteriorly or posteriorly) on kinematics and kinetics during a maximal sprint. | 20-m | 3% AWR/ 3% PWR | WR was placed 2/3 of the load on the thigh and the remaining 1/3 on the shank (anteriorly or posteriorly) | ↑ 10-20 m ST for WR conditions; ↑ CT for WR conditions in the start and acceleration phase; ↓ SF for WR conditions in the start phase | CT_(UL x AWR Start)_: 3.5%/≤0.05*/NA  CT_(UL x PWR Start)_: 4.5%/≤0.05*/NA  CT_(AWR x PWR Start)_:  0.97%/NA/NA  CT_(UL x AWR Accel.)_: 3.14%/≤0.05*/NA  CT_(UL x PWR Accel.)_: 3.14%/≤0.05*/NA  CT_(AWR x PWR Accel.)_: 0%/NA/NA  FT_(UL x AWR Start)_: -7.27%/≥0.05/NA  FT_(UL x PWR Start)_: -7.27%/≥0.05/NA  FT_(AWR x PWR Start)_: 0%/NA/NA  FT_(UL x AWR Accel.)_: 1.27%/≥0.05/NA  FT_(UL x PWR Accel.)_: 2.53%/≥0.05/NA  FT_(AWR x PWR Accel.)_: 1.25%/NA/NA  SL_(UL x AWR Start)_: 0%/≥0.05/NA  SL_(UL x PWR Start)_: 0.83%/≥0.05/NA  SL_(AWR x PWR Start)_: 0.82%/NA/NA  SL_(UL x AWR Accel.)_: 0%/≥0.05/NA  SL_(UL x PWR Accel.)_: 0%/≥0.05/NA  SL_(AWR x PWR Accel.)_:0%/NA/NA  SF_(UL x AWR Start)_: -1.27%/≥0.05/NA  SF_(UL x PWR Start)_: -1.27%/≥0.05/NA  SF_(AWR x PWR Start)_: 0%/NA/NA  SF_(UL x AWR Accel.)_: -2.37%/≤0.05*/NA  SF_(UL x PWR Accel.)_: -2.6%/≤0.05*/NA  SF_(AWR x PWR Accel.)_: -0.24%/NA/NA | ST_(UL x AWR 2-m)_: -1.2%/≥0.05/NA  ST_(UL x PWR 2-m)_: -1.2%/≥0.05/NA  ST_(AWR x PWR 2-m)_: 0%/NA/NA  ST_(UL x AWR 5-m)_: 0%/≥0.05/NA  ST_(UL x PWR 5-m)_: 0%/≥0.05/NA  ST_(AWR x PWR 5-m)_: 0%/NA/NA  ST_(UL x AWR 10-m)_: 0%/≥0.05/NA  ST_(UL x PWR 10-m)_: 0.45%/≥0.05/NA  ST_(AWR x PWR 10-m)_: 0.45%/NA/NA  ST_(UL x AWR 20-m)_: 0.84%/≥0.05/NA  ST_(UL x PWR 20-m)_: 1.4%/≥0.05/NA  ST_(AWR x PWR 20-m)_: 0.56%/NA/NA  ST_(UL x AWR 10-20-m)_: 2.2%/≤0.05*/NA  ST_(UL x PWR 10-20-m)_: 3%/≤0.05*/NA  ST_(AWR x PWR 10-20-m)_: 0.72%/NA/NA |
| Macadam et al. ^17^ | Athletes performed 2 sprints in each condition (WR, UL). | To determine the acute changes of forearm WR in kinematics and kinetics during sprint-running. | 20-m | 2% | Evenly distributed on the forearm | ↑ WR 10/20/10-20 m ST; ↓ FT, SF in WR condition; ↑ CT, SL in WR condition. | CT_(UL x WR)_: 7.1%/<0.01*/0.73  FT_(UL x WR)_: 6%/0.01*/-0.22  SL_(UL x WR)_: 1.82%/0.22/0.04  SF_(UL x WR)_: -4.22%/<0.01*/-0.67 | ST_(UL x WR 2-m)_: 1.70%/0.23/0.39  ST_(UL x WR 5-m)_: 2.68%/0.08/0.48  ST_(UL x WR 10-m)_: 2.14%/0.02*/0.46  ST_(UL x WR 20-m)_: 2.17%/<0.01*/0.43  ST_(UL x WR 10-20 m)_: 2.20%/0.01*/0.33 |
| Simperingham et al. ^13^ | Athletes performed 3 sets of 2 30-m sprint repetition on a track and on a non-motorized treadmill (separate days) under 3 different conditions (UL; WR 3%; WR 5%). | To quantify the kinematic and kinetic changes with 3 to 5% WR during sprinting. | 30-m sprint on a track and on a non-motorized treadmill | 3/5% | WR load evenly distributed around the thigh and shank with 2/3 of the load anteriorly and 1/3 of the load posteriorly | ↑ 20-m ST in 5% WR compared to UL and 3% WR; ↑ CT in 3 and 5% WR compared to UL in the start phase; ↑ CT and ↓ SF in 3 and 5% WR compared to UL in the acceleration phase. | CT_(UL x WR 3% Start)_: 4.57%/≤0.05*/0.41-0.49  CT_(UL x WR 5% Start)_: 5.08%/≤0.05*/0.41-0.49  CT_(WR 3% x WR 5% Start)_: 0.48%/>0.05/NA  CT_(UL x WR 3% Accel.)_: 4.46%/≤0.05*/0.56  CT_(WR 5% x UL Accel.)_: 5.73%/≤0.05*/0.72  CT_(WR 3% x WR 5% Accel.)_: 1.22%/>0.05/NA  FT_(UL x WR 3% Start)_: - 19.35%/>0.05/NA  FT_(UL x WR 5% Start)_: - 17.74%/>0.05/NA  FT_(WR 3% x WR 5% Start)_: 2%/>0.05/NA  FT_(UL x WR 3% Accel.)_: -3.75%/>0.05/NA  FT_(UL x WR 5% Accel.)_: - 3.75%/>0.05/NA  FT_(WR 3% x WR 5% Accel.)_: 0%/>0.05/NA  SL_(UL x WR 3% Start)_: 0.82%/>0.05/NA  SL_(UL x WR 5% Start)_: 0.0%/>0.05/NA  SL_(WR 3% x WR 5% Start)_: -0.81%/>0.05/NA  SL_(UL x WR 3% Accel.)_: 0.0%/>0.05/NA  SL_(UL x WR 5% Accel.)_: -0.62%/>0.05/NA  SL_(WR 3% x WR 5% Accel.)_: 0.62%/>0.05/NA  SF_(UL x WR 3% Start)_: -1.50%/>0.05/NA  SF_(UL x WR 5% Start)_: -2.00%/>0.05/NA  SF_(WR 3% x WR 5% Start)_: -0.51%/>0.05/NA  SF_(UL x WR 3% Accel.)_: -1.65%/≤0.05*/0.32  SF_(UL x WR 5% Accel.)_:-2.59%/≤0.05*/0.52  SF_(WR 5% x WR 3% Accel.)_:-0.96%/>0.05/NA | ST_(5-m UL x WR 3%)_: -1.48%/>0.05/NA  ST_(5-m UL x WR 5%)_: 0.74%/>0.05/NA  ST_(5-m WR 3% x WR 5%)_: 2.26%/>0.05/NA  ST_(10-m UL x WR 3%)_: - 0.47%/>0.05/NA  ST_(10-m UL x WR 5%)_: 0.94%/>0.05/NA  ST_(10-m WR 3% x WR 5%)_: 1.42%/>0.05/NA  ST_(20-m UL x WR 3%)_: 0.58%/>0.05/NA  ST_(20-m UL x WR 5%)_: 2.02%/≤0.05*/0.38  ST_(20-m WR 3% x WR 5%)_: 1.44%/≤0.05*/0.27 |
| Uthoff et al. ^21^ | Athletes performed 2 maximum efforts sprints under forearm WR condition and 1 maximum effort sprint under UL condition. | To determine the acute changes of forearm WR in kinematic and kinetic during sprinting. | 30-m | 2% | Evenly placed around the forearm. | ↓ SF in WR condition in phases 2 and 3 of the sprint compared to UL | CT_(UL x WR phase 1)_: 5.51%/>0.05/0.65  CT_(UL x WR phase 2)_: 4.04%/>0.05/0.43  CT_(UL x WR phase 3)_: 1.68%/>0.05/0.28  CT_(UL x WR phase 4)_: 1.76%/>0.05/0.31  FT_(UL x WR phase 1)_: -1.33%/>0.05/-0.10  FT_(UL x WR phase 2)_: 4.53%/>0.05/0.46  FT_(UL x WR phase 3)_: 5.74%/>0.05/0.60  FT_(UL x WR phase 4)_: 5.28%/>0.05/0.55  SL_(UL x WR phase 1)_: 3.73%/>0.05/0.40  SL_(UL x WR phase 2)_: 2.91%/>0.05/0.47  SL_(UL x WR phase 3)_: 3.14%/>0.05/0.52  SL_(UL x WR phase 4)_: 2.94%/>0.05/0.45  SF_(UL x WR phase 1)_: -3.09%/>0.05/-0.60  SF_(UL x WR phase 2)_: -3.42%/<0.05*/-0.81  SF_(UL x WR phase 3)_: -3.60%/<0.05*/-0.86  SF_(UL x WR phase 4)_: -3.68%/>0.05/-0.72 | ST_(10-m UL x WR)_: 1.80%/>0.05/-0.32  ST_(30-m UL x WR)_: 0.87/>0.05/-0.24 |
| Uthoff et al. ^36^ | Athletes performed 2 maximum efforts sprints under forearm WR condition and 1 maximum effort sprint under UL condition. | To determine the acute changes of forearm WR in kinematic and kinetic during sprinting. | 30-m | 2% | Evenly placed around the forearm. | ↑ SL in WR condition in phase 1 compared to UL; ↓ SF and ↑ FT in WR condition in phase 4 compared to UL | CT_(UL x WR phase 1)_: 4.80%/>0/05/0.56  CT_(UL x WR phase 2)_: 3.79%/>0.05/0.63  CT_(UL x WR phase 3)_: 2.63%/>0.05/0.42  CT_(UL x WR phase 4)_: 2.61%/>0.05/0.45  FT_(UL x WR phase 1)_: -0.90%/>0.05/-0.05  FT_(UL x WR phase 2)_: 4.19%/>0.05/0.43  FT_(UL x WR phase 3)_: 6.76%/>0.05/0.67  FT_(UL x WR phase 4)_: 7.48%/≤0.05*/0.79  SL_(UL x WR phase 1)_: 3.97%/≤0.05*/1.04  SL_(UL x WR phase 2)_: 2.94%/>0.05/0.46  SL_(UL x WR phase 3)_: 3.68%/>0.05/0.56  SL_(UL x WR phase 4)_: 3.94%/>0.05/0.57  SF_(UL x WR phase 1)_: -1.69%/>0.05/-0.45  SF_(UL x WR phase 2)_: -3.87%/>0.05/-0.81  SF_(UL x WR phase 3)_: -4.50%/>0.05/-0.86  SF_(UL x WR phase 4)_: -4.82%/≤0.05*/-0.92 | ST_(10-m UL x WR)_: 1.38%/>0.05/-0.54  ST_(30-m UL x WR)_: 1.54%/>0.05/-0.38  ST_(10-30 m UL x WR)_: 1.69%/>0.05/-0.50 |
| Zhang et al. ^14^ | Athletes performed three maximal speed sprints under each of the 2 different conditions (WR and UL) | To determine the effects of added mass on the shank on the sprinting technique. | 65-m | ~0.55% of the sample BM (15% of the shank mass) | Adjusted bag around the shank (WR) | ↑ CT in WR condition compared to UL. | CT_(UL x WR)_: 10%/<0.01*/NA  SL_(UL x WR)_: -0.45%/0.66/NA  SF_(UL x WR)_: -2.08%/0.21/NA |  |

Abbreviations: Accel., Acceleration phase; AV, Average; AWR, Anterior wearable resistance; BM, Body mass; CT, Ground contact time; ES, Effect Size; FT, Flight time; F-V, Force-velocity; NA, Not available; PWR, Posterior wearable resistance; SF, Step frequency; SL, Step length; ST, Sprint time; UL, Unloaded; WR, Wearable resistance.

*Indicate significant difference (<0.05).

Eletronic Supplementary Material Table S2 – Study characteristics for WV cross-over interventions.

| Study | Intervention | Study objective | Sprint Test | WR/WV Load (%BM) | WR/WV Placement | WR/WV Sprint Performance (velocity and time)/Kinematic Results | WR/WV Sprint Kinematic Results (%/p/ES) | WR/WV Sprint Performance  (%/p/ES) |
| --- | --- | --- | --- | --- | --- | --- | --- | --- |
| Carlos-Vivas et al. ^11^ | Athletes competed 10 all-out sprints under 5 different conditions (2 trials per load) (UL, WV 10%, WV 20%, WV 30% and WV 40%). | To describe the load velocity relationship and the effects on spatiotemporal and kinetics variables using different weights of WV in running. | 30-m | 10/20/30/40% | WV | ↑ ST when load increased | NA | ST_(30-m UL x WV 10%)_: 4.32%/<0.001*/Moderate  ST_(30-m UL x WV 20%)_: 7.99%/<0.001*/Large  ST_(30-m UL x WV 30%)_: 11.88%/<0.001*/Very large  ST_(30-m UL x WV 40%)_: 16.63%/<0.001*/Very large  ST_(30-m WV 10% x WV 20%)_: 3.52%/<0.001*/Moderate  ST_(30-m WV 10% x WV 30%)_: 7.25%/<0.001*/Large  ST_(30-m WV 10% x WV 40%)_: 11.80%/<0.001*/Very large  ST_(30-m WV 20% x WV 30%)_: 3.6%/<0.001*/Moderate  ST_(30-m WV 20% x WV 40%)_: 8%/<0.001*/Large  ST_(30-m WV 30% x WV 40%)_: 4.25%/<0.001*/Moderate |
| Cronin et al. ^28^ | Athletes performed 5 30-m sprint under 5 different conditions (UL, WV 15/20% and Sled 15/20%). | To determine the effects of WV of sled towing on performance and kinematics. | 30-m | 15/20% | WV with a belt around the waist attached to the shoulders with straps | ↓ ST for both WV conditions compared to UL; ↓ ST for WV 20% compared to WV 15% condition; ↓ SL for both WV conditions compared to UL; ↓ SF for both WV conditions compared to UL; ↑ Swing Ph in UL compared to WV; ↑ Stance Ph for both WV conditions compared to UL; ↑ Stance Ph for WV 20% condition compared to WV 15% condition | SL_(5-m UL x 15%)_: -6.71%/≤0.05*/NA  SL_(5-m UL x 20%)_: -9.76%/≤0.05*/NA  SL_(5-m WV 15% x WV 20%)_: -3.27%/>0.05/NA  SL_(15-m UL x 15%)_: -5.02%/≤0.05*/NA  SL_(15-m UL x 20%)_: -7.03%/≤0.05*/NA  SL_(15-m WV 15% x WV 20%)_: -2.12%/>0.05/NA  SL_(25-m UL x 15%)_: -7.66%/≤0.05*/NA  SL_(25-m UL x 20%)_: -10.81%/≤0.05*/NA  SL_(25-m WV 15% x WV 20%)_: -3.41%/>0.05/NA  SF_(5-m UL x 15%)_: -4.66%/≤0.05*/NA  SF_(5-m UL x 20%)_: -4.89%/≤0.05*/NA  SF_(5-m WV 15% x WV 20%)_: -0.24%/>0.05/NA  SF_(15-m UL x 15%)_: -2.76%/≤0.05*/NA  SF_(15-m UL x 20%)_: -3.46%/≤0.05*/NA  SF_(15-m WV 15% x WV 20%)_: -0.71%/>0.05/NA  SF_(25-m UL x 15%)_: -3.69%/≤0.05*/NA  SF_(25-m UL x 20%)_: -4.85%/≤0.05*/NA  SF_(25-m WV 15% x WV 20%)_: -1.20%/>0.05/NA  Stance Ph _(5-m UL x 15%)_: 16.67%/≤0.05*/NA  Stance Ph _(5-m UL x 20%)_: 18.94%/≤0.05*/NA  Stance Ph _(5-m WV 15% x WV 20%)_: 1.95%/≤0.05*/NA  Stance Ph _(15-m UL x 15%)_: 13.11%/≤0.05*/NA  Stance Ph _(15-m UL x 20%)_: 17.21%/≤0.05*/NA  Stance Ph _(15-m WV 15% x WV 20%)_: 3.62%/≤0.05*/NA  Stance Ph _(25-m UL x 15%)_: 18.01%/≤0.05*/NA  Stance Ph _(25-m UL x 20%)_: 24.32%/≤0.05*/NA  Stance Ph _(25-m WV 15% x WV 20%)_: 5.34%/≤0.05*/NA  Swing Ph _(5-m UL x 15%)_: -9.71%/≤0.05*/NA  Swing Ph _(5-m UL x 20%)_: -14.56%/≤0.05*/NA  Swing Ph _(5-m WV 15% x WV 20%)_: -5.38%/>0.05/NA  Swing Ph _(15-m UL x 15%)_: -8.26%/≤0.05*/NA  Swing Ph _(15-m UL x 20%)_: -11.93%/≤0.05*/NA  Swing Ph _(15-m WV 15% x WV 20%)_: -4%/>0.05/NA  Swing Ph _(25-m UL x 15%)_: -9.09%≤0.05*/NA  Swing Ph _(25-m UL x 20%)_: -12.40%≤0.05*/NA  Swing Ph _(25-m WV 15% x WV 20%)_: -3.64/>0.05/NA | ST_(10-m UL x WV 15%)_: 7.56%/≤0.05*/NA  ST_(10-m UL x WV 20%)_: 9.88%/≤0.05*/NA  ST_(10-m WV 15% x WV 20%)_: 2.16%/>0.05/NA  ST_(30-m UL x WV 15%)_: 9.49%/≤0.05*/NA  ST_(30-m UL x WV 20%)_: 11.43%/≤0.05*/NA  ST_(30-m WV 15% x WV 20%)_: 1.78%/≤0.05*/NA |
| Cross et al. ^12^ | Athletes completed 6 seconds sprints under UL condition and 2 different WV loads. | To analyze the effects of WV on kinetics and kinematics during sprinting. | 6-second maximum sprint | 10.9/21.8% | WV with loads evenly distributed around the torso. | ↓ SL in both vest conditions compared to UL; ↑ CT in both vest conditions during maximum velocity phase compared to UL;  ↓ FT in 21.8% WV during acceleration phase; ↓ FT in both vest conditions during maximum velocity phase compared to UL | CT_(Accel. UL x WV 10.9%)_: -4.05%/>0.05/NA  CT_(Max. vel. UL x WV 10.9%)_: 5.92%/<0.05*/1.01-1.71  CT_(Accel. UL x WV 21.8%)_: 1.35%/>0.05/NA  CT_(Max. vel. UL x WV 21.8%)_: 10.06%/<0.05*/1.01-1.71  CT_(Accel. WV 10.89 x WV 21.8%)_: 5.63%/>0.05/NA  CT_(Max. vel. WV 10.89 x WV 21.8%):_ 3.91%/>0.05/NA  FT_(Accel. UL x WV 10.9%)_: -20%/>0.05/NA  FT_(Max. vel. UL x WV 10.9%)_: -17.39%/<0.05*/-0.89-(-1.08)  FT_(Accel. UL x WV 21.8%)_: -27.00%/<0.05*/-1.50  FT_(Max. vel. WV 21.8% x UL)_: -18.84%/<0.05*/-0.89-(-1.08)  FT_(Accel. WV 10.9% x WV 21.8%)_: -8.33%/>0.05/NA  FT_(Max. vel. WV 10.9% x WV 21.8%)_: -1.75%/>0.05/NA  SL_(Max. vel. UL x WV 10.9%)_: -4.22%/<0.05*/-0.33-(-0.34)  SL_(Max. vel. UL x WV 21.8%)_: -4.22%/<0.05*/-0.33-(-0.34)  SL_(WV 10.9% x WV 21.8%)_: 0%/>0.05/NA  SF_(UL x WV 10.9%)_: 0.72%/>0.05/NA  SF_(UL x WV 21.8%)_: - 1.45%/>0.05/NA  SF_(WV 10.9% x WV 21.8%)_: -2.15%/>0.05/NA | NA |
| Gleadhill et al. ^30^ | Athletes completed one maximum effort 60-m sprint under 2 different conditions (WV, UL) | To analyze the differences in kinetic and kinematic with WV or UL conditions during sprinting. | 60-m | 7% | WV trunk mounted with loads evenly distributed around the torso | ↑ Support time in initial and middle in WV condition. | SL_(initial UL x WV)_: -2.72%/>0.05/-0.36  SL_(middle UL x WV)_: -2.47%/>0.05/-0.48  SL_(later UL x WV)_: -2.32%/>0.05/-0.53  SL_(Max. vel. UL x WV)_: -1.95%/>0.05/-0.43  FT_(initial accel. UL x WV)_: -8.45%/>0.05/-0.41  FT_(middle accel. UL x WV)_: -3.85%/>0.05/-0.47  FT_(later accel. UL x WV)_: -1.68%/>0.05/-0.29  FT_(Max. vel. UL x WV)_: -1.67%/>0.05/-0.28  SF_(initial accel. UL x WV)_: -0.86%/>0.05/-0.17  SF_(middle accel. UL x WV)_: -1.38%/>0.05/-0.30  SF_(later accel. UL x WV)_: -1.55%/>0.05/-0.34  SF_(Max. vel. UL x WV)_: - 1.51%/>0.05/-0.32  SuT_(initial accel. UL x WV)_: 4.88%/<0.05*/0.76  SuT _(middle accel. UL x WV)_: 6.09%/<0.05*/0.85  SuT _(later accel. UL x WV)_: 6.00%/>0.05/0.74  SuT _(Max. vel. UL x WV)_: 5.05%/>0.05/0.70 | NA |
| Zafeiropoulos et al. ^37^ | Athletes performed 2 sprint tests under each of the 4 different conditions (UL, WV 8%, WV 15%, WV 20%). | To examine the acute effects of different WV loads, on performance of a sprint. | 50-m flying start sprint | 8%/15%/ 20% | WV | ↑ 10/20/30/40/50/10-20/20-30/30-40-m ST under 8% WV compared with UL; ↑ 10/20/30/40/50/10-20/20-30/30-40-m ST under 15% WV compared with UL; ↑ 10/20/30/40/50/10-20/20-30/30-40-m ST under 20% WV compared with UL; ↑ 20-30/30-40-m ST under 20% WV compared with 8% WV | NA | ST_(10-m UL x WV 8%)_: 4.07%/<0.05*/NA  ST_(20-m UL x WV 8%)_: 4.67%/<0.05*/NA  ST_(10-20m UL x WV 8%)_: 5.47%/<0.05*/NA  ST_(30-m UL x WV 8%)_: 4.62%/<0.05*/NA  ST_(20-30m UL x WV 8%)_:  5.30%/<0.05*/NA  ST_(40-m UL x WV 8%)_: 5.11%/<0.05*/NA  ST_(30-40m UL x WV 8%)_:  9.56%/<0.05*/NA  ST_(50-m UL x WV 8%)_: 4.65%/<0.05*/NA  ST_(40-50m UL x WV 8%)_:  2.52%/>0.05/NA  ST_(10-m UL x WV 15%)_: 6.98%/<0.05*/NA  ST_(20-m UL x WV 15%)_: 7.33%/<0.05*/NA  ST_(10-20m UL x WV 15%)_: 7.81%/<0.05*/NA  ST_(30-m UL x WV 15%)_: 6.24%/<0.05*/NA  ST_(20-30m UL x WV 15%)_: 6.82%/<0.05*/NA  ST_(40-m UL x WV 15%)_: 7.48%/<0.05*/NA  ST_(30-40m UL x WV 15%)_: 9.56%/<0.05*/NA  ST_(50-m UL x WV 15%)_: 6.75%/<0.05*/NA  ST_(40-50m UL x WV 15%)_: 3.36%/>0.05/NA  ST_(10-m UL x WV 20%)_: 9.88%/<0.05*/NA  ST_(20-m UL x WV 20%)_: 9.33%/<0.05*/NA  ST_(10-20m UL x WV 20%)_: 8.59%/<0.05*/NA  ST_(30-m UL x WV 20%)_: 9.24/<0.05*/NA  ST_(20-30m UL x WV 20%)_: 9.85%/<0.05*/NA  ST_(40-m UL x WV 20%)_: 9.85%/<0.05*/NA  ST_(30-40m UL x WV 20%)_: 12.17%/<0.05*/NA  ST_(50-m UL x WV 20%)_: 8.85%/<0.05*/NA  ST_(40-50m UL x WV 20%)_: 4.20%/>0.05/NA  ST_(10-m WV 8% x WV 15%)_: 2.79%/>0.05/NA  ST_(20-m WV 8% x WV 15%)_: 2.55%/>0.05/NA  ST_(10-20m WV 8% x WV 15%)_: 2.22%/>0.05/NA  ST_(30-m WV 8% x WV 15%)_: 1.54%/>0.05/NA  ST_(20-30m WV 8% x WV 15%)_: 1.44%/>0.05/NA  ST_(40-m WV 8% x WV 15%)_: 2.26%/>0.05/NA  ST_(30-40m WV 8% x WV 15%)_: 0%/>0.05/NA  ST_(50-m WV 8% x WV 15%)_: 2.01%/>0.05/NA  ST_(40-50m WV 8% x WV 15%)_: 0.82%/>0.05/NA  ST_(10-m WV 8% x WV 20%)_: 5.59%/>0.05/NA  ST_(20-m WV 8% x WV 20%)_: 4.46%/>0.05/NA  ST_(10-20m WV 8% x WV 20%)_: 2.96%/>0.05/NA  ST_(30-m WV 8% x WV 20%)_: 4.41/>0.05/NA  ST_(20-30m WV 8% x WV 20%)_: 4.32%/<0.05*/NA  ST_(40-m WV 8% x WV 20%)_: 4.51%/>0.05/NA  ST_(30-40m WV 8% x WV 20%)_: 2.38%/<0.05*/NA  ST_(50-m WV 8% x WV 20%)_: 4.01%/>0.05/NA  ST_(40-50m WV 8% x WV 20%)_: 1.64%/>0.05/NA  ST_(10-m WV 15% x WV 20%)_: 2.71%/>0.05/NA  ST_(20-m WV 15% x WV 20%)_: 1.86%/>0.05/NA  ST_(10-20m WV 15% x WV 20%)_: 0.72%/>0.05/NA  ST_(30-m WV 15% x WV 20%)_: 2.83%/>0.05/NA  ST_(20-30m WV 15% x WV 20%)_: 2.84%/>0.05/NA  ST_(40-m WV 15% x WV 20%)_: 2.21/>0.05/NA  ST_(30-40m WV 15% x WV 20%)_: 2.38%/>0.05/NA  ST_(50-m WV 15% x WV 20%)_: 1.97%/>0.05/NA  ST_(40-50m WV 15% x WV 20%)_: 0.81%/>0.05/NA |

Abbreviations: Accel., Acceleration phase; BM, Body mass; CT, Ground contact time; ES, Effect Size; FT, Flight time; Max.vel., Maximum velocity phase; NA, Not available; SF, Step frequency; SL, Step length; ST, Sprint time; SuT, Support time; Swing Ph, Swing phase; UL, Unloaded; WV, Weighted vest.

*Indicate significant difference (<0.05).

Eletronic Supplementary Material Table S3 – Study characteristics for WR longitudinal intervention.

| Study | Intervention | Study objective | Sprint Test | WR/WV Load (%BM) | WR/WV Placement | WR/WV Sprint Performance (velocity and time)/Kinematic Results | WR/WV Sprint Kinematic Results (%;ES/p) | WR/WV Sprint Performance  (%/p/ES) | Training protocol |
| --- | --- | --- | --- | --- | --- | --- | --- | --- | --- |
| Feser et al. ^15^ | Athletes completed performance and kinetic tests before and after 6 weeks of 2 dedicated sprint training per week along with sport specific training under WR or UL intervention | To determine the effects of a 6-week WR intervention on sprint running time and kinetics variables. | 30-m | 1% | Attached to the shank. | Pre and post WR group did not presented any significant change in all variables; Pre and post UL group showed significant ↑ in 5/10/20/30-m time; ↓ 5/10/20-m ST in WR condition compared to UL. | NA | ST_(5-m WR pre x post)_: 1.54%/0.38/0.29  ST_(10-m WR pre x post)_: 0.48%/0.42/0.13  ST_(20-m WR pre x post)_: 0.30%/0.60/0.08  ST_(30-m WR pre x post)_: 0.22%/0.77/0.06  ST_(5-m UL pre x post)_: 7.87%/<0.01*/1.25  ST_(10-m UL pre x post)_: 4.90%/0.01*/0.91  ST_(20-m UL pre x post)_: 3.60%/0.02*/0.63  ST_(30-m UL pre x post)_: 2.20%/0.05*/0.36  ST_(5-m pre UL x WR)_: 2.36%/>0.05/NA  ST_(10-m pre UL x WR)_: 1.47%/>0.05/NA  ST_(20-m pre UL x WR)_: 1.20%/>0.05/NA  ST_(30-m pre UL x WR)_: 0.66%/>0.05/NA  ST_(5-m post UL x WR)_: -3.65%/0.01*/1.17  ST_(10-m post UL x WR)_: -2.80%/0.02*/1.03  ST_(20-m post UL x WR)_: -2.03%/0.05*/0.89  ST_(30-m post UL x WR)_: -1.29%/0.11/0.71 | 1^ST^ Week Session  Week 1  4S x 22-m  8S x 10-m  Week 2  5S x 22-m  11S x 10-m  Week 3  6S x 22-m  14S x 10-m  Week 4  5S x 22-m  11S x 10-m  Week 5  6S x 22-m  13S x 10-m  Week 6  6S x 22-m  16S x 10-m  2^ND^ Week Session  Week 1  4 × Flying 28 m 5 × Change of direction (15 m-diagonal cut-20 m) 1 × 80 m, 1 × 60 m, 1 × 50 m, 1 × 40 m  Week 2  Training session cancelled  Week 3  5 × Flying 28 m 8 × Change of direction (15 m-diagonal cut-20 m) 1 × 80 m, 1 × 60 m, 1 × 50 m, 1 × 40 m  Week 4  5 × Flying 28 m 6 × Change of direction (15 m-diagonal cut-20 m) 1 × 80 m, 1 × 60 m, 1 × 50 m, 1 × 40 m  Week 5  5 × Flying 28 m 8 × Change of direction (15 m-diagonal cut-20 m) 1 × 80 m, 1 × 60 m, 1 × 50 m, 1 × 40 m  Week 6  5 × Flying 28 m 9 × Change of direction (15 m-diagonal cut-20 m) 1 × 80 m, 1 × 60 m, 1 × 50 m, 1 × 40 m |
|  |  |  |  |  |  |  |  |  |  |

Abbreviations: BM, Body mass; ES, Effect size; NA, Not available; pre, Pre intervention; post, Post Intervention; S, Sets; ST, Sprint time; UL, Unloaded; WR, Wearable resistance.

*Indicate significant difference (<0.05).

Eletronic Supplementary Material Table S4 – Study characteristics for WV longitudinal interventions.

| Study | Intervention | Study objective | Sprint Test | WR/WV Load (%BM) | WR/WV Placement | WR/WV Sprint Performance (velocity and time)/Kinematic Results | WR/WV Sprint Kinematic Results (%;p/ES) | WR/WV Sprint Performance  (%/p/ES) | Training protocol |
| --- | --- | --- | --- | --- | --- | --- | --- | --- | --- |
| Barr et al. ^10^ | Athletes completed performance, kinematic and jump tests before and after 8-day of rugby practices, speed training, and strength training sessions under 2 conditions (simulated hypergravity for the WV group or UL group). | To examine if 8-day of simulated hypergravity was effective at changing the sprinting speed and lower-body power. | 40-m | 12% | NA | ↓ CT in acceleration and maximal velocity phase with WV. | CT_(WV Accel. pre x post)_: 0%/>0.05/0.28  CT_(UL Accel. pre x post)_: 0%/>0.05/0.18  CT_(pre Accel. UL x WV)_: -5.88%/NA/NA  CT_(post Accel. UL x WV)_: - 5.88%/NA/NA  CT_(WV Max. vel. pre x post)_: -9.09%/**0.03**/0.09  CT_(UL Max. vel. pre x post)_: 0%/>0.05/0.1  CT_(pre Max. vel. UL x WV)_: 0%/NA/NA  CT_(post Max. vel. UL x WV)_: -9.09%/NA/NA  FT_(WV Accel. pre x post)_: 0%/>0.05/0.25  FT_(UL Accel. pre x post)_: 0%/>0.05/0.14  FT_(pre Accel. UL x WV)_: 0%/NA/NA  FT_(post Accel. UL x WV)_: 0%/NA/NA  FT_(WV Max. vel. pre x post)_: 9.09%/>0.05/0.54  FT_(UL Max. vel. pre x post)_: 9.09%/>0.05/0.4  FT_(pre Max. vel. UL x WV)_: 0%/NA/NA  FT_(post Max. vel. UL x WV)_: 0%/NA/NA  SL_(WV Accel. pre x post)_: 0.80%/>0.05/0.07  SL_(UL Accel. pre x post)_: 1.50%/>0.05/0.18  SL_(pre Accel. UL x WV)_: 6.01%/NA/NA  SL_(post Accel. UL x WV)_: 6.67%/NA/NA  SL_(WV Max. vel. pre x post)_: 2.47%/>0.05/0.28  SL_(UL Max. vel. pre x post)_: 2.41%/>0.05/0.57  SL_(pre Max. vel. UL x WV)_: - 2.41%/NA/NA  SL_(post Max. vel. UL x WV)_: 2.36%/NA/NA | ST(_0-10m WV pre x post)_: 0%/>0.05/0  ST(_0-10m UL pre x post)_: 0%/>0.05/0  ST_(0-10m pre UL x WV)_: 0%/NA/NA  ST_(0-10m post UL x WV)_: 0%/NA/NA  ST(_30-40m WV pre x post)_: 0%/>0.05/0.02  ST(_30-40m UL pre x post)_: 0%/>0.05/0  ST_(30-40m pre WV x UL)_: 0%/NA/NA  ST_(30-40m post WV x UL)_: 0%/NA/NA  ST(_40-m WV pre x post)_: 0%/>0.05/0.17  ST(_40-m UL pre x post)_: -0.19%/>0.05/0.03  ST_(40-m pre UL x WV)_: -0.95%/NA/NA  ST_(40-m post UL x WV)_: -1.14%/NA/NA | Rugby practices, speed training (200-m volume per session ranging from 10-m and 25-m sprint) and strength training (5-6 sets, 2-8 reps). |
| Clark et al. ^18^ | Athletes completed performance and kinematic tests before and after 7 weeks of 2 sprint training per week (except for week 6) under 3 different conditions (WV, WSL, UL). | To determine and compare their effectiveness the longitudinal effects of resisted sprint training using WSL or WV for improving sprint performance. | 36.6-m (18.3- to 54.9-m). | 18.5% | 0.45 kg weights evenly distributed around the vest. | No significant differences were noted between groups for performance and kinematics. | CT_(WV pre x post)_: -1.53%/>0.05/0.25  CT_(UL pre x post)_: -3.79%/>0.05/0.38  CT_(pre UL x WV)_: -0.76%/>0.05/NA  CT_(post UL x WV)_: 1.57%/>0.05/NA  FT_(WV pre x post)_: -5.40%/>0.05/1.00  FT_(UL pre x post)_: 0%/>0/05/0  FT_(pre UL x WV)_: 6.73%/>0.05/NA  FT_(post UL x WV)_: 0.96%/>0.05/NA | ST_(WV pre x post)_: - 1.20%/>0.05/0.25  ST_(UL pre x post)_: -1.97%/>0.05/0.61  ST_(pre UL x WV)_: 0.85%/>0.5/NA  ST_(post UL x WV)_: 1.63%/>0.05/NA | 1^ST^ Week Session  Week 1: 3R x 18.3-m  2R x 36.6-m  2R x 54.9-m  Week 2: 3R x 18.3-m  3R x 36.6-m  2R x 54.9-m  Week 3: 3R x 18.3-m  3R x 36.6-m  3R x 54.9-m  Week 4: 2R x 18.3-m  4R x 36.6-m  2R x 54.9-m  Week 5: 2R x 18.3-m  4R x 36.6-m  4R x 54.9-m  Week 6: 2R x 18.3-m  2R x 36.6-m  2R x 54.9-m  Week 7: 3R x 18.3-m  3R x 36.6-m  2R x 54.9-m  2^ND^ Week Session  Week 1: 2R x 54.9-m  2R x 36.6-m  3R x 18.3-m  Week 2: 2R x 54.9-m  3R x 36.6-m  3R x 18.3-m  Week 3: 3R x 54.9-m  3R x 36.6-m  3R x 18.3-m  Week 4: 3R x 54.9-m  4R x 36.6-m  2R x 18.3-m  Week 5: 4R x 54.9-m  4R x 36.6-m  2R x 18.3-m  Week 6: No training  Week 7: 3R x 54.9-m  3R x 36.6-m  3R x 18.3-m |
| Rey et al. ^19^ | Athletes performed 6-weeks of sprint training, 2 times a week, combined with their training routine, with pre- and post-performance evaluations. | To determine the effects of a 6-week, program using WVs as compared with traditional UL training on jump, sprint, and RSA performance. | 10- and 30-m | 18.9% | WV | ↓ 10-/30-m pre- to post- ST in WV and UL group. | NA | ST_(10-m UL pre x post)_: -11.17%/<0.001*/2.50  ST_(30-m UL pre x post)_: -5.15%/<0.001*/1.86  ST_(10-m WV pre x post)_: -9.55%/<0.001*/1.77  ST_(30-m WV pre x post)_: -5.99%/<0.001*/3.30  ST_(10-m pre UL x WV)_: -0.56%/>0.05/NA  ST_(30-m pre UL x WV)_: 1.64%/>0.05/NA  ST_(10-m post UL x WV)_: 1.26%/>0.05/NA  ST_(30-m post UL x WV)_: 0.74%/>0.05/NA | Week 1: 1S x 6R x 20-m  Week 2: 2S x5R x 20-m  Week 3: 2S x 5R x 20-m  Week 4: 4S x 3R x 20-m  Week 5: 4S x 3R x 20-m  Week 6: 2S x 7R x 20-m |
| Simpson et al. ^35^ | Participants in the WV group were instructed to wear a WV 4 days a week for 8 hours and for 3 training sessions a week during 21 days. Thereafter, WV were instructed to remove the WV from their routine for more 21 days. UL group continue their normal routine training. Both groups were evaluated pre-, 21 days and 42 days after. | To assess performance measures following 3 weeks of WV routine in well-trained females. | 25-m | 8% | WV | No differences were found in baseline and group x time interactions 25-m ST. | NA | ST_(25-m WV pre x post)_: -0.96%/>0.05/NA  ST_(25-m UL pre x post)_: -0.95%/>0.05/NA  ST_(25-m pre UL x WV)_: -1.19%/>0.05/NA  ST_(25-m post UL x WV)_: -1.20%/>0.05/NA | WV worn WV in 4-days a week during 8-hours period and in 3 training sessions for 21 days. |

Abbreviations: Accel., Acceleration phase; BM, Body mass; CT, Ground contact time; ES, Effect size; FT, Flight time; Max.vel., Maximum velocity phase; NA, Not available; pre, Pre intervention; post, Post Intervention; R, Repetition; S, Sets; SL, Step length; ST, Sprint time; UL, Unloaded; WSL, Weighted sled; WV, Weighted vest.

*Indicate significant difference (<0.05).

Eletronic Supplementary Material Table S5 – PRISMA Cheklist

| **Section and Topic** | **Item #** | **Checklist item** | **Location where item is reported** |
| --- | --- | --- | --- |
| **TITLE** | | |  |
| Title | 1 | Identify the report as a systematic review. | 1 |
| **ABSTRACT** | | |  |
| Abstract | 2 | See the PRISMA 2020 for Abstracts checklist. | 1 |
| **INTRODUCTION** | | |  |
| Rationale | 3 | Describe the rationale for the review in the context of existing knowledge. | 1-2 |
| Objectives | 4 | Provide an explicit statement of the objective(s) or question(s) the review addresses. | 2 |
| **METHODS** | | |  |
| Eligibility criteria | 5 | Specify the inclusion and exclusion criteria for the review and how studies were grouped for the syntheses. | 2 |
| Information sources | 6 | Specify all databases, registers, websites, organisations, reference lists and other sources searched or consulted to identify studies. Specify the date when each source was last searched or consulted. | 2 |
| Search strategy | 7 | Present the full search strategies for all databases, registers and websites, including any filters and limits used. | 2 |
| Selection process | 8 | Specify the methods used to decide whether a study met the inclusion criteria of the review, including how many reviewers screened each record and each report retrieved, whether they worked independently, and if applicable, details of automation tools used in the process. | 2 |
| Data collection process | 9 | Specify the methods used to collect data from reports, including how many reviewers collected data from each report, whether they worked independently, any processes for obtaining or confirming data from study investigators, and if applicable, details of automation tools used in the process. | 2 |
| Data items | 10a | List and define all outcomes for which data were sought. Specify whether all results that were compatible with each outcome domain in each study were sought (e.g. for all measures, time points, analyses), and if not, the methods used to decide which results to collect. | 2 |
|  | 10b | List and define all other variables for which data were sought (e.g. participant and intervention characteristics, funding sources). Describe any assumptions made about any missing or unclear information. | 2 |
| Study risk of bias assessment | 11 | Specify the methods used to assess risk of bias in the included studies, including details of the tool(s) used, how many reviewers assessed each study and whether they worked independently, and if applicable, details of automation tools used in the process. | 2 |
| Effect measures | 12 | Specify for each outcome the effect measure(s) (e.g. risk ratio, mean difference) used in the synthesis or presentation of results. | 2-3 |
| Synthesis methods | 13a | Describe the processes used to decide which studies were eligible for each synthesis (e.g. tabulating the study intervention characteristics and comparing against the planned groups for each synthesis (item #5)). | 2-3 |
|  | 13b | Describe any methods required to prepare the data for presentation or synthesis, such as handling of missing summary statistics, or data conversions. | 2-3 |
|  | 13c | Describe any methods used to tabulate or visually display results of individual studies and syntheses. | 2-3 |
|  | 13d | Describe any methods used to synthesize results and provide a rationale for the choice(s). If meta-analysis was performed, describe the model(s), method(s) to identify the presence and extent of statistical heterogeneity, and software package(s) used. | 2-3 |
|  | 13e | Describe any methods used to explore possible causes of heterogeneity among study results (e.g. subgroup analysis, meta-regression). | 2-3 |
|  | 13f | Describe any sensitivity analyses conducted to assess robustness of the synthesized results. | 2-3 |
| Reporting bias assessment | 14 | Describe any methods used to assess risk of bias due to missing results in a synthesis (arising from reporting biases). | NA |
| Certainty assessment | 15 | Describe any methods used to assess certainty (or confidence) in the body of evidence for an outcome. | NA |
| **RESULTS** | | |  |
| Study selection | 16a | Describe the results of the search and selection process, from the number of records identified in the search to the number of studies included in the review, ideally using a flow diagram. | 3 |
|  | 16b | Cite studies that might appear to meet the inclusion criteria, but which were excluded, and explain why they were excluded. | 3 |
| Study characteristics | 17 | Cite each included study and present its characteristics. | 3 |
| Risk of bias in studies | 18 | Present assessments of risk of bias for each included study. | 3 |
| Results of individual studies | 19 | For all outcomes, present, for each study: (a) summary statistics for each group (where appropriate) and (b) an effect estimate and its precision (e.g. confidence/credible interval), ideally using structured tables or plots. | 3-10 |
| Results of syntheses | 20a | For each synthesis, briefly summarise the characteristics and risk of bias among contributing studies. | 3-10 |
|  | 20b | Present results of all statistical syntheses conducted. If meta-analysis was done, present for each the summary estimate and its precision (e.g. confidence/credible interval) and measures of statistical heterogeneity. If comparing groups, describe the direction of the effect. | 3-10 |
|  | 20c | Present results of all investigations of possible causes of heterogeneity among study results. | 3-10 |
|  | 20d | Present results of all sensitivity analyses conducted to assess the robustness of the synthesized results. | 3-10 |
| Reporting biases | 21 | Present assessments of risk of bias due to missing results (arising from reporting biases) for each synthesis assessed. | NA |
| Certainty of evidence | 22 | Present assessments of certainty (or confidence) in the body of evidence for each outcome assessed. | NA |
| **DISCUSSION** | | |  |
| Discussion | 23a | Provide a general interpretation of the results in the context of other evidence. | 10-12 |
|  | 23b | Discuss any limitations of the evidence included in the review. | 13 |
|  | 23c | Discuss any limitations of the review processes used. | 13 |
|  | 23d | Discuss implications of the results for practice, policy, and future research. | 12 |
| **OTHER INFORMATION** | | |  |
| Registration and protocol | 24a | Provide registration information for the review, including register name and registration number, or state that the review was not registered. | NA |
|  | 24b | Indicate where the review protocol can be accessed, or state that a protocol was not prepared. | NA |
|  | 24c | Describe and explain any amendments to information provided at registration or in the protocol. | NA |
| Support | 25 | Describe sources of financial or non-financial support for the review, and the role of the funders or sponsors in the review. | 14 |
| Competing interests | 26 | Declare any competing interests of review authors. | 14 |
| Availability of data, code and other materials | 27 | Report which of the following are publicly available and where they can be found: template data collection forms; data extracted from included studies; data used for all analyses; analytic code; any other materials used in the review. | NA |
